# Vision-language models for medical report generation and visual question answering: a review

**DOI:** 10.3389/frai.2024.1430984

**Published:** 2024-11-19

**Authors:** Iryna Hartsock, Ghulam Rasool

**Affiliations:** Department of Machine Learning, H. Lee Moffitt Cancer Center and Research Institute, Tampa, FL, United States

**Keywords:** vision-language models, report generation, visual question answering, datasets, evaluation metrics, healthcare

## Abstract

Medical vision-language models (VLMs) combine computer vision (CV) and natural language processing (NLP) to analyze visual and textual medical data. Our paper reviews recent advancements in developing VLMs specialized for healthcare, focusing on publicly available models designed for medical report generation and visual question answering (VQA). We provide background on NLP and CV, explaining how techniques from both fields are integrated into VLMs, with visual and language data often fused using Transformer-based architectures to enable effective learning from multimodal data. Key areas we address include the exploration of 18 public medical vision-language datasets, in-depth analyses of the architectures and pre-training strategies of 16 recent noteworthy medical VLMs, and comprehensive discussion on evaluation metrics for assessing VLMs' performance in medical report generation and VQA. We also highlight current challenges facing medical VLM development, including limited data availability, concerns with data privacy, and lack of proper evaluation metrics, among others, while also proposing future directions to address these obstacles. Overall, our review summarizes the recent progress in developing VLMs to harness multimodal medical data for improved healthcare applications.

## 1 Introduction

The last decade has seen significant progress in artificial intelligence (AI) and machine learning (ML), including the development of foundation models (FMs), large language models (LLMs), and vision-language models (VLMs). These AI/ML developments have started transforming several aspects of our daily lives, including healthcare. AI/ML can potentially transform the healthcare continuum by significantly optimizing and improving disease screening, diagnostics, treatment planning, and post-treatment care (Bajwa et al., 2021). Various computer vision (CV) and natural language processing (NLP) models, particularly LLMs, have been instrumental in driving this transformative trend (He et al., 2023b; Zhou et al., 2023b). CV models have been trained and validated for various screening and diagnosis use cases leveraging radiology data from X-rays, mammograms, magnetic resonance imaging (MRI), computed tomography (CT), and others. Recently, AI models focused on digital pathology using histopathology and immunohistochemistry data have also shown significant advances in accurate disease diagnosis, prognosis, and biomarker identification (Waqas et al., 2023, 2024a). On the other hand, by training models using large datasets of medical literature, clinical notes, and other healthcare-related text, LLMs can extract insights from electronic health records (EHR) efficiently, assist healthcare professionals in generating concise summary reports, and facilitate the interpretation of patient information. Noteworthy examples of such LLMs include *GatorTron* (Yang et al., 2022), *ChatDoctor* (Li et al., 2023c), *Med-PaLM* (Medical Pathways Language Model; Singhal et al., 2023), and *Med-Alpaca* (Han et al., 2023).

The healthcare data is inherently multimodal, and consequently, the AI/ML models often need to be trained using multiple data modalities, including text (e.g., clinical notes, radiology reports, surgical pathology reports, etc.), imaging (e.g., radiology scans, digitized histopathology slides, etc.), and tabular data (e.g., numerical data such as vitals or labs and categorical data such as race, gender, and others; Acosta et al., 2022; Shrestha et al., 2023; Waqas et al., 2024b; Tripathi et al., 2024a; Mohsan et al., 2023; Waqas et al., 2024c,a; Tripathi et al., 2024b). In routine clinical practice, healthcare professionals utilize a combination of these data modalities for diagnosing and treating various conditions. Integrating information from diverse data modalities enhances the precision and thoroughness of disease assessments, diagnoses, treatment planning, and post-treatment surveillance. The need for AI/ML models to ingest, integrate, and learn from information stemming from varied data sources is the driving force for *multimodal learning* (Huang et al., 2021; Waqas et al., 2024b).

The recent progress in multimodal learning has been driven by the development of VLMs (Gan et al., 2022; Chen et al., 2023; Mohsan et al., 2023). These models analyze, interpret, and derive insights from both visual and textual data. In the medical domain, these models contribute to a holistic understanding of patient information and improve ML model performance in clinical tasks. Many of these models, like *CLIP* (Contrastive Language—Image Pre-training; Radford et al., 2021), *LLaVa* (Large Language and Vision Assistant; Liu et al., 2023c), and *Flamingo* (Alayrac et al., 2022) are tailored to healthcare domain through training on extensive medical datasets. Adapting VLMs for medical visual question-answering (VQA; Lin et al., 2023b) enables healthcare professionals to query medical images such as CT scans, MRIs, mammograms, ultrasounds, X-rays, and more. The question-answering capability elevates the interactive nature of the AI/ML models in healthcare, facilitating dynamic exchanges between healthcare providers and the AI system. Furthermore, adapting VLMs for medical report generation enables them to amalgamate information from visual and textual sources, producing detailed and contextually relevant reports. This enhances healthcare workflow efficiency by ensuring comprehensive and accurate reports.

In contrast to previous related surveys (Lin et al., 2023b; Ting et al., 2023; Shrestha et al., 2023), this review aims to provide a comprehensive update on how methods from CV and NLP are integrated to develop VLMs specifically designed for medical report generation and VQA. The specific objectives of this review are as follows:

- Provide essential background on artificial neural networks, CV, and NLP, to ensure the accessibility of this review for readers from medical fields and promote collaboration and knowledge exchange between the AI/ML community and the medical professionals (see Section 2).
- Explore the integration of CV and NLP in VLMs, including model architectures, training strategies, and downstream tasks (see Section 3).
- Analyze recent advances in VLMs, datasets, and evaluation metrics relevant to medical report generation and VQA (see Section 4). Specifically:
  - Describe 18 publicly available vision-language datasets that encompass medical image-text pairs or question-answer pairs related to medical images (see Section 4.1).
  - Outline over 10 metrics employed for evaluating VLMs in the context of report generation and VQA tasks (see Section 4.2).
  - Thoroughly review 16 recent medical VLMs, 15 of which are publicly available, with most models not previously covered in other surveys (see Section 4.2).
- Discuss the current challenges within the field of medical VLMs, offering insights into potential research directions that could profoundly influence their future development (see Section 5).

**Figure 1:**
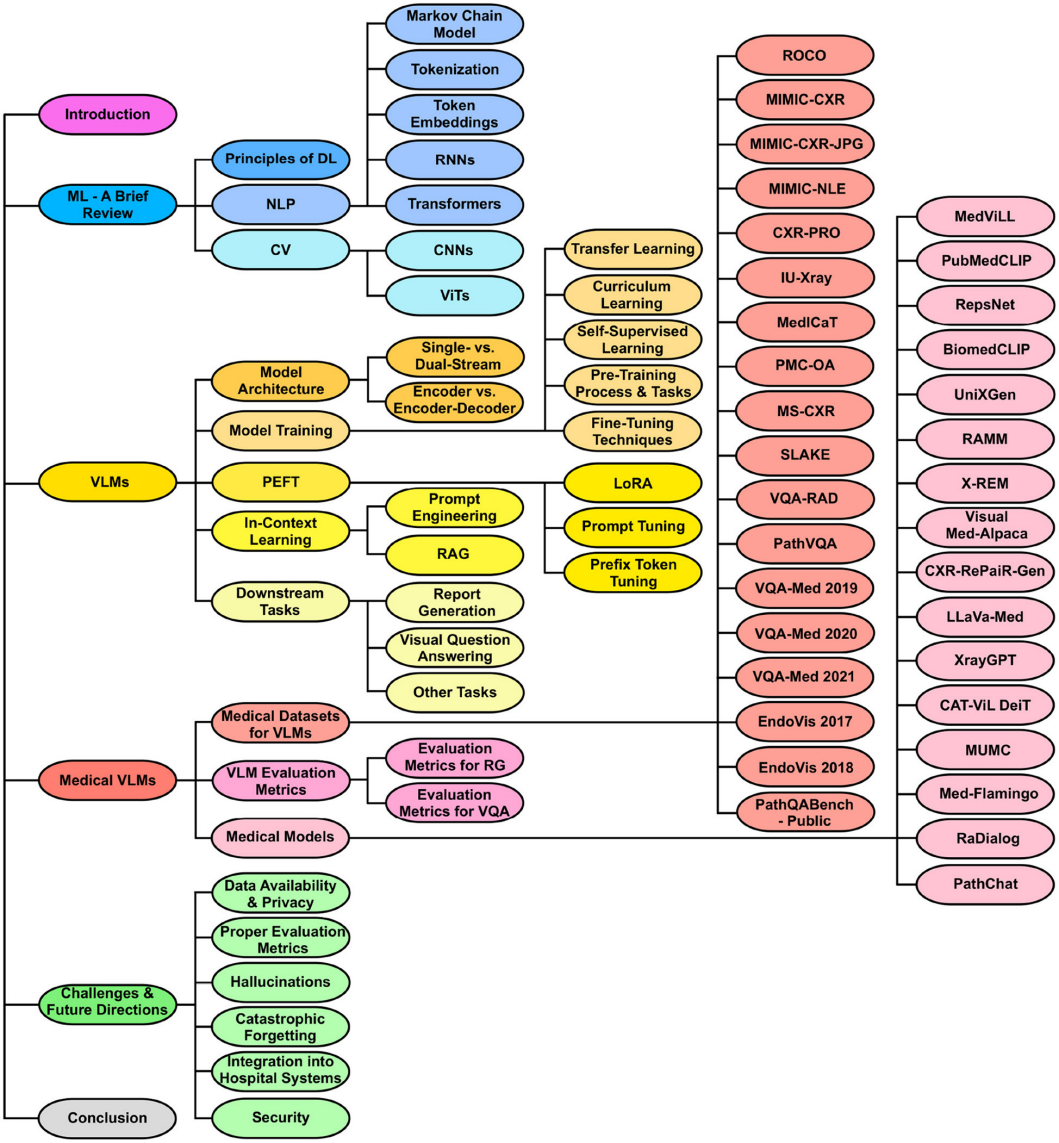
Organization of the review paper. The structure begins with an introduction, followed by a foundational review of ML and background on VLMs. It then delves into medical vision-language datasets, evaluation metrics, and recent medical VLMs. Next, the paper addresses the current challenges of medical VLMs and proposes possible future research directions. It ends with a conclusion summarizing key insights and findings.

The overall structure of this review is shown in Figure 1. The list of medical VLMs and datasets can also be found on: GitHub.

## 2 Machine learning—a brief review

Deep learning (DL), a subfield of ML, involves algorithms that learn to recognize patterns and make decisions by analyzing large amounts of data. In this section, we review the fundamental principles of DL and explore two main areas of DL relevant to medical VLMs: CV and NLP. For more detailed information on DL, we refer the reader to LeCun et al. (2015), Goodfellow et al. (2016), and Baldi (2021).

### 2.1 Principles of deep learning

ML and AI originated in the 1940–1950's, with neural networks (NNs) emerging as classical models. The fundamental building block of an NN is an artificial neuron, which receives multiple inputs, aggregates them, applies nonlinear operations, and outputs a single scalar value. NNs consist of layers of interconnected artificial neurons, including input, output, and hidden layers. In feedforward NNs, connections are structured so that a connection from neuron *i* to neuron *j* exists only if *i*<*j* (Baldi, 2021). In any NN, the connections between artificial neurons carry weight, and neurons utilize “activation functions” on their inputs to introduce non-linearity. An activation function is a mathematical operation that transforms the weighted sum of inputs into an output, enabling the network to model complex patterns. Common activation functions include the sigmoid, hyperbolic tangent (tanh), and Rectified Linear Unit (ReLU).

A loss function quantifies the disparity between predicted and actual outputs, with the goal of minimizing this scalar value during training. DL leverages NNs but extends them into deeper architectures with many hidden layers. Backpropagation, short for backward propagation of errors, is essential for training deep NNs. It involves calculating the gradient of the loss function with respect to the weights, using the chain rule for derivatives (Baldi, 2021). This gradient information updates the weights to minimize the loss. Common optimization methods include gradient descent, stochastic gradient descent (SGD; Robbins, 1951), and Adam (Adaptive Moment Estimation; Kingma and Ba, 2014). These methods iteratively update the weights to improve the model's performance during training.

### 2.2 Natural language processing

NLP is the analysis of linguistic data, most commonly in the form of textual data such as documents or publications, using computational methods (Verspoor and Cohen, 2013). NLP encompasses a variety of tasks aimed at understanding, processing, and generating human language. The common NLP tasks include machine translation, named entity recognition, text summarization, etc. In the following, we introduce terminology and fundamental concepts that will help the reader in the coming sections on modern NLP and medical VLMs.

#### 2.2.1 Markov chain model

The Markov chain model has historically been significant in NLP, particularly for tasks involving sequence prediction and probabilistic modeling of text data (Nadkarni et al., 2011). A Markov chain is a stochastic process that transitions from one state to another based on specific probabilistic rules, with the fundamental property that the future state depends only on the current state and not on the sequence of events that preceded it. This property, known as the Markov property, allowed Markov chains to model the likelihood of sequences of words or characters by capturing statistical dependencies between adjacent elements. They facilitated tasks such as text generation, next-element prediction, and part-of-speech tagging in early NLP research and applications, providing a foundational framework for subsequent advanced techniques (Nadkarni et al., 2011).

#### 2.2.2 Tokenization

In contemporary NLP, tokenization is the initial step involving the splitting of sentences and words into their smallest morphemes, known as tokens (Rai and Borah, 2021). Subword tokenization methods are often preferred in many NLP applications due to their effectiveness in handling out-of-vocabulary words. *WordPiece* (Wu et al., 2016) starts by treating each character as a token, forming an initial vocabulary. Using a flexible merging strategy, WordPiece considers adjacent characters or subword units that enhance the overall likelihood of the training data, aiming to accurately represent it given the model's current state. *Byte-Pair Encoding* (BPE; Sennrich et al., 2016) shares similarities with WordPiece but follows a more deterministic merging strategy. BPE merges the most frequent pair of adjacent characters or subword units in each iteration, progressing toward a predefined vocabulary size. *Byte-level BPE* (Wang et al., 2020) operates at an even finer granularity, considering individual bytes instead of characters. This extension allows it to capture more nuanced patterns at the byte level.

#### 2.2.3 Token embeddings

Tokens are often transformed into numerical vectors that capture semantic relationships between tokens, called word or token embeddings. *Word2Vec* (Mikolov et al., 2013b) is a widely used word embedding technique employing two models: Skip-Gram (Mikolov et al., 2013b) and Continuous Bag of Words (CBOW; Mikolov et al., 2013a). Skip-Gram predicts context words given a target word, capturing semantic associations, while CBOW predicts the target word based on context, emphasizing syntactic structures. Word2Vec is computationally efficient, making it suitable for large datasets and general-purpose applications. *Global Vectors* (GloVe; Pennington et al., 2014) focuses on capturing global semantic relationships by analyzing word pair statistics across the entire corpus. It generates word vectors reflecting co-occurrence probabilities, which is ideal for tasks requiring a holistic understanding of word connections. *FastText* (Bojanowski et al., 2017) is effective for handling out-of-vocabulary words and morphologically rich languages. It adopts a sub-word approach, breaking words into n-grams, and uses a skip-gram training method similar to Word2Vec to learn embeddings for these sub-word units.

Specialized embeddings are available for biomedical and clinical terms. *BioWordVec* (Zhang et al., 2019) incorporates MeSH terms and text from PubMed abstracts to learn improved biomedical word embeddings. *Cui2vec* (Beam et al., 2020) utilizes multi-modal data from medical publications and clinical notes, mapping terms onto a common Concept Unique Identifier (CUI) space. Additionally, *positional encodings*, often based on sinusoidal functions, are commonly added to capture the order of tokens in a sequence. These vectors systematically encode token positions, enriching embeddings with positional information for tailored NLP tasks (Ahmed et al., 2023).

#### 2.2.4 Recurrent neural networks

RNNs are widely employed for pattern detection in sequential data like genomic sequences, text, or numerical time series (Schmidt, 2019). Operating on the principle of preserving a form of memory, RNNs incorporate a cyclic structure by looping the output of a specific layer back to the input, facilitating the prediction of subsequent layer outputs. This mechanism empowers RNNs to adeptly model sequential and temporal dependencies, capturing information from preceding time steps within hidden states. However, they face challenges in retaining long-term dependencies due to the vanishing gradient problem. To address this, variants like Long Short-Term Memory (LSTM; Hochreiter and Schmidhuber, 1997) and Gated Recurrent Unit (GRU; Cho et al., 2014) have been developed to better capture and utilize long-range dependencies in sequential data (Ahmed et al., 2023).

#### 2.2.5 Transformers

In recent years, there has been a remarkable advancement in NLP mainly due to the development of the Transformer models (Vaswani et al., 2017). Beyond incorporating embeddings and positional encodings, the Transformer architecture consists of an encoder that processes input data, represented by vectors obtained from embedded and positionally encoded tokens. The encoder-generated representation then serves as the input for the subsequent decoder, transforming these vector representations into a relevant output tailored to the specific task. A defining characteristic of the Transformer lies in its *self-attention* mechanism, particularly the scaled dot-product attention, which proves instrumental in capturing intricate dependencies within sequences.

The synergy between enhanced computational power provided by Graphical Processing Units (GPUs) and advancements in attention mechanisms has been pivotal in developing large language models (LLMs). These models are meticulously trained on vast datasets with many parameters. BERT (Bidirectional Encoder Representations from Transformers; Devlin et al., 2019) marked the inception of LLMs. The era of even larger LLMs began in 2020 with the introduction of models like GPT-3 (the 3rd generation of the Generative Pre-trained Transformer model; Brown et al., 2020) and PaLM (Pathways Language Model; Chowdhery et al., 2022). Some recent LLMs include LLaMA (Large Language Model Meta AI; Touvron et al., 2023a,b), Vicuna (Chiang et al., 2023), and Mistral (Jiang et al., 2023).

### 2.3 Computer vision

CV involves interpreting and understanding the world from their images or videos (Ji, 2020). Data in CV is encoded as numerical values representing the intensity or brightness of pixels. The extraction of visual patterns like edges, textures, and objects in images or video frames serves as building blocks for various CV tasks like image classification, object detection, and semantic segmentation. In the following, we introduce fundamental concepts and terms essential for understanding VLMs presented in the later parts of the paper.

#### 2.3.1 Convolutional neural networks

CNNs represent a significant advancement in CV (Yamashita et al., 2018). Besides pooling and fully connected layers, CNNs also have convolution layers, which apply convolution operations to input data. A small filter or kernel slides over the input data during a convolution operation, performing element-wise multiplications with local regions of the input at each position. The results are summed to create a new value in the output feature map. This process is repeated across the entire input, capturing patterns and features at different spatial locations. The well-known CNNs include Residual Network (ResNet; He et al., 2016), Dense Convolutional Network (DenseNet; Huang et al., 2022), Efficient Network (EfficientNet; Tan and Le, 2020), and others.

#### 2.3.2 Vision transformers

Transformer models, originally proposed for NLP tasks, have also found valuable applications in CV. For instance, the ViT model (Dosovitskiy et al., 2021) can capture intricate relationships and dependencies across the entire image. This is achieved by leveraging the Transformer architecture and treating images as sequences of smaller patches. Each image patch undergoes flattening into a vector, followed by passage through an embedding layer, enriching the patches for a more expressive representation. Positional encodings are then incorporated to convey spatial arrangement information. ViTs also introduce a special token capturing global image information, represented by a learnable token embedding with unique parameters. ViTs have excelled in semantic segmentation (Ranftl et al., 2021), anomaly detection (Mishra et al., 2021), medical image classification (Manzari et al., 2023; Barhoumi et al., 2023), and even outperformed CNNs in some cases (Tyagi et al., 2021; Xin et al., 2022).

## 3 Vision-language models

Many real-world scenarios inherently involve multiple data modalities, prompting the development of VLMs capable of simultaneously handling and understanding both NLP and CV data. In this section, we build on the basic concepts described earlier and present VLMs, their architectures, training and fine-tuning methods, and various downstream tasks facilitated by these multimodal models.

### 3.1 Model architecture

#### 3.1.1 Single-stream vs. dual-stream VLMs

Based on how different data modalities are fused together in VLMs, they are generally categorized into two groups (Chen et al., 2023): (1) *single-stream* (e.g., VisualBERT; Li et al., 2019 and UNITER or UNiversal Image-TExt Representation Learning; Chen et al., 2020b), and (2) *dual-stream* models (e.g., ViLBERT or Vision-and-Language BERT; Lu et al., 2019 and CLIP or Contrastive Language-Image Pre-training; Radford et al., 2021).

**Figure 2:**
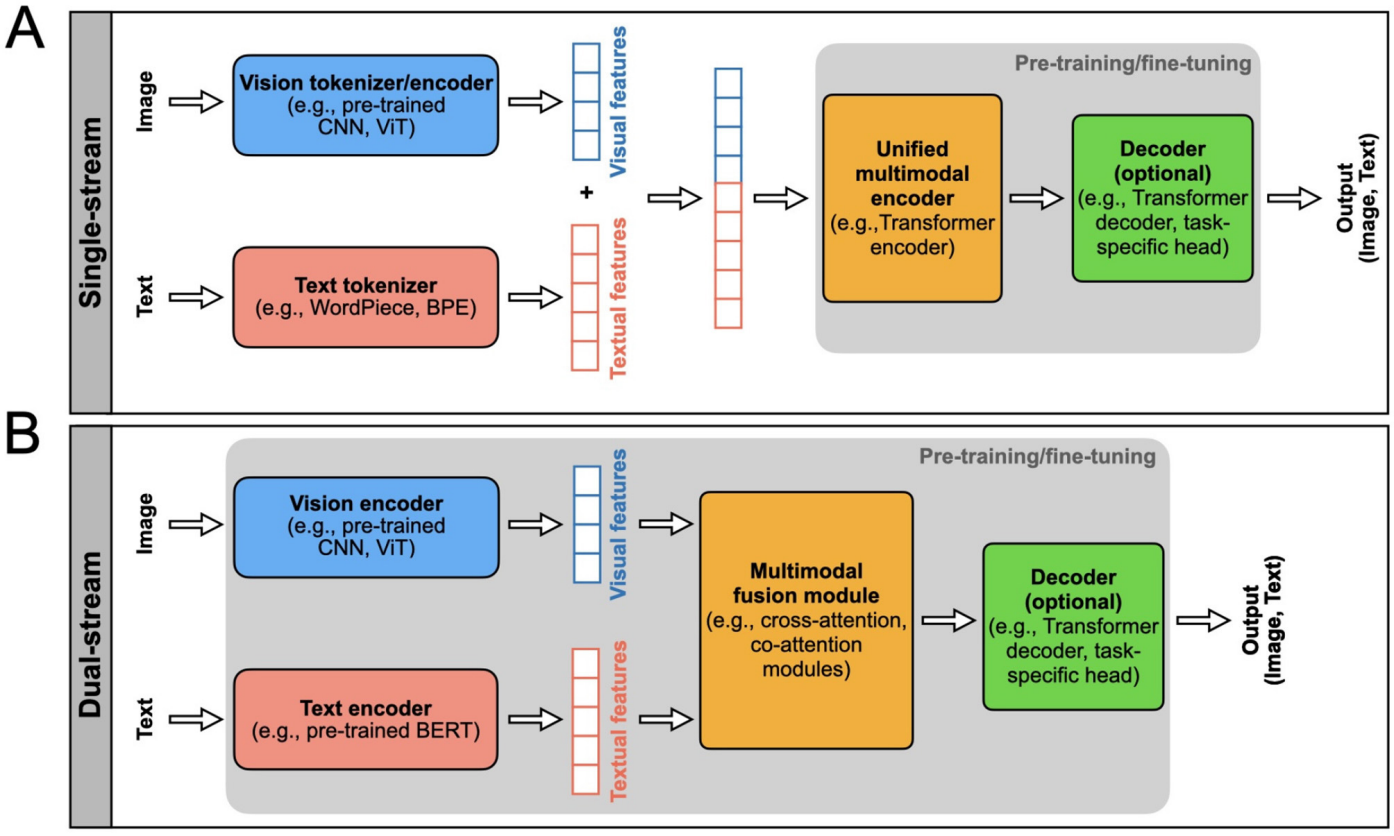
Two main types of VLM architectures, single-steam and dual-stream, are presented. The model inputs and outputs are indicated. The rectangular boxes inside the gray areas indicate the components of the VLM that typically undergo pre-training and fine-tuning, i.e., the model parameters are updated using labeled or unlabeled data. The top row **(A)** shows the single-stream VLM architecture, and the bottom row shows the **(B)** dual-stream. Each block indicated in these architectures can be designed using different AI/ML models as indicated in these blocks.

**Figure 3:**
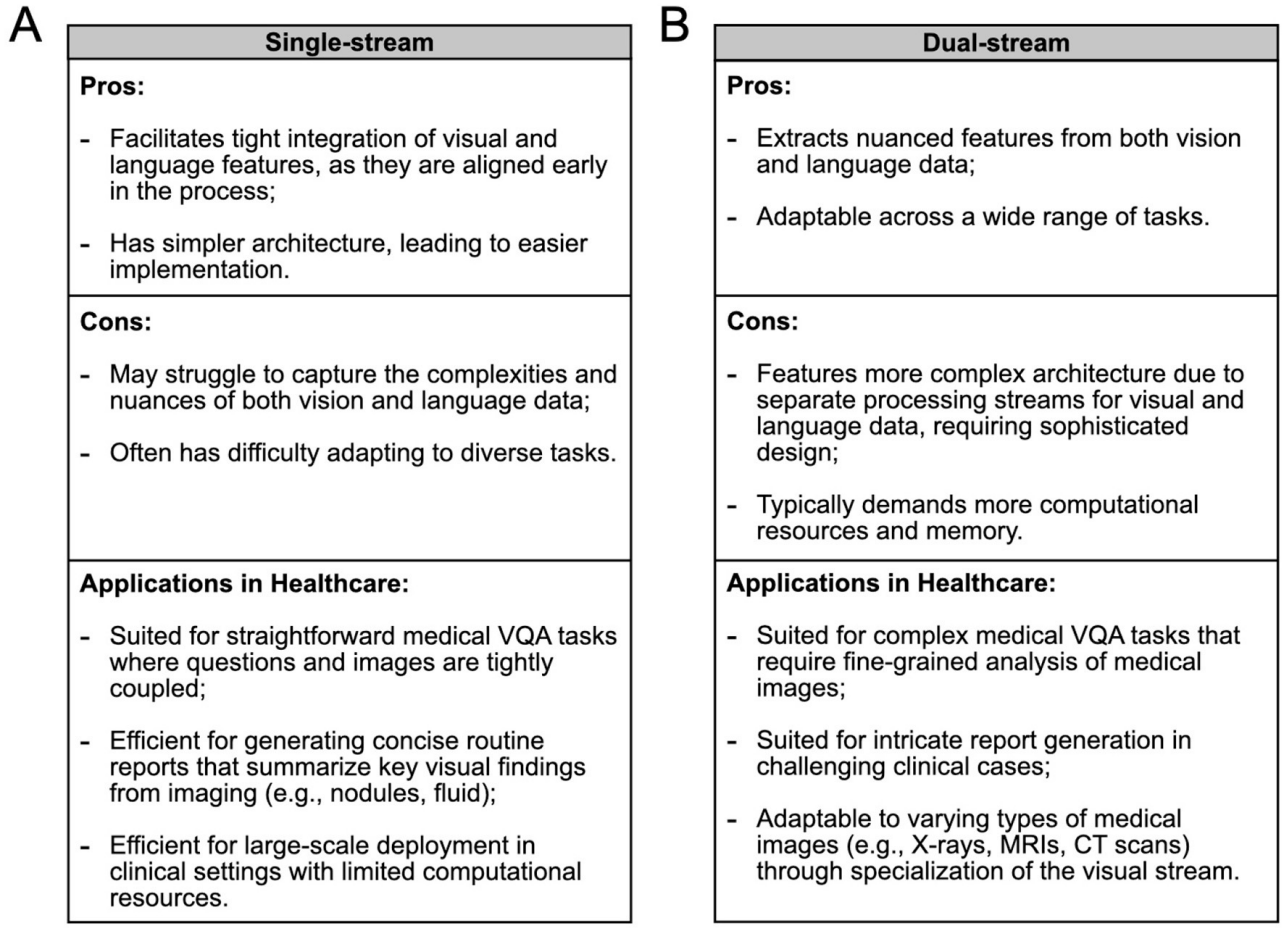
Comparison of **(A)** single-stream and **(B)** dual-stream VLMs in terms of their advantages, disadvantages, and healthcare applications, to guide the selection of the appropriate architecture for various medical scenarios. In some cases, the optimal choice between architectures remains uncertain and may depend on specific task requirements.

A **single-stream** VLM adopts an efficient architecture for processing visual and textual information within a unified module (see Figure 2A and Figure 3A). This architecture incorporates an early fusion of distinct data modalities, concatenating feature vectors from various data sources into a single vector (e.g., MedViLL; Moon et al., 2022). Subsequently, this combined representation is fed into a single stream. One notable advantage of the single-stream design is its parameter efficiency, achieved by employing the same set of parameters for all modalities. This simplifies the model and contributes to computational efficiency during training and inference phases (Chen et al., 2023).

A **dual-stream** VLM extracts visual and textual representations separately in parallel streams without parameter sharing (see Figure 2B and Figure 3B). This architecture typically exhibits higher computational complexity than single-stream architectures. Visual features are generated from pre-trained *vision encoders*, such as CNNs or ViTs, and textual features are obtained from pre-trained *text encoders*, usually based on the Transformer architecture (e.g., PubMedCLIP; Eslami et al., 2023). These features are then integrated using a *multimodal fusion module*, often leveraging attention mechanisms, to capture cross-modal dependencies.

#### 3.1.2 Encoder vs. encoder-decoder VLMs

The learned cross-modal representations can be optionally processed by a *decoder* before producing the final output. Consequently, VLMs are classified into two groups: (1) *encoder-only* [e.g., ALIGN (A Large-scale ImaGe and Noisy-text embedding; Jia et al., 2021)] and (2) *encoder-decoder* models [e.g., SimVLM (Simple Visual Language Model; Wang et al., 2022c)].

**Encoder-only** VLMs are advantageous in scenarios where the primary objective is efficient representation learning. They often exhibit streamlined processing and reduced computational complexity, making them suitable for tasks requiring compact and informative representations. However, these models might lack the capability to generate intricate and detailed outputs, limiting their use in tasks demanding nuanced responses or creative generation.

**Encoder-decoder** VLMs offer the flexibility to generate complex and diverse outputs, making them well-suited for tasks like image captioning, translation, or any application requiring creative responses. The decoding step allows for the transformation of joint representations into meaningful outputs. However, this versatility comes at the cost of increased computational demand and complexity.

### 3.2 Model training

#### 3.2.1 Transfer learning

A widely used strategy in ML is transfer learning, where pre-trained models are customized for specific downstream tasks. This involves fine-tuning the model's parameters using smaller task-specific datasets to address the intricacies of the target task rather than starting with random initialization (Bommasani et al., 2022). Transfer learning often entails modifying the original model's architecture, such as adjusting final layers or introducing new ones, like classification or regression layers, to align with the task requirements (Bommasani et al., 2022). The goal is to adapt the pre-trained model to the new task while leveraging the knowledge it gained during initial pre-training. Almost all VLMs use transfer learning during training in one way or another.

#### 3.2.2 Curriculum learning

Curriculum learning offers a novel approach for tasks or data with inherent progressions or hierarchies. It strategically presents training examples or tasks in a designed order, often based on difficulty or complexity measures (Soviany et al., 2021). For instance, LLaVa-Med, a recent medical VLM (Li et al., 2023a), employs curriculum learning during training. This gradual learning approach starts with simpler examples and progresses to more complex ones, enhancing the model's adaptability and performance.

#### 3.2.3 Self-supervised learning

SSL provides a potent alternative to traditional supervised learning by enabling models to generate their own labels from data (Rani et al., 2023). This approach is especially advantageous when acquiring labeled data is difficult or costly. In self-supervised learning for VLMs, models formulate tasks that leverage inherent data structures, allowing them to learn meaningful representations across modalities without external labels. Examples of such tasks include contrastive learning, masked language modeling, and masked image modeling (further detailed in the subsequent sub-section).

#### 3.2.4 Pre-training process and tasks

The pre-training process is crucial for providing VLMs with a foundational understanding of the complex relationship between visual and textual data. A common approach involves extensive pre-training on datasets pairing images/videos with their corresponding textual descriptions. Throughout pre-training, the model engages in various tasks to acquire versatile representations for downstream applications. The following paragraphs describe commonly used pre-training techniques.

**Contrastive learning (CL)** trains the model to distinguish positive pairs from negative pairs of visual and textual data (Li et al., 2021). Positive pairs contain related visual and textual content, like an image with its corresponding description. Negative pairs contain unrelated content, such as an image paired with a randomly chosen description. The goal is to bring positive pairs closer and push negative pairs apart in a shared embedding space. Various contrastive loss functions are used, with InfoNCE (Noise-Contrastive Estimation) loss (van den Oord et al., 2019) being a common choice. CLIP (Radford et al., 2021) employs InfoNCE with cosine similarity, while ALIGN (Jia et al., 2021) uses normalized softmax loss to enhance positive similarity and reduce negative similarities.

**Masked language modeling (MLM)** is an NLP task (Taylor, 1953) first utilized in BERT (Devlin et al., 2019). MLM randomly replaces a percentage of tokens in textual data with a special token, usually denoted as MASK. The model then predicts these masked tokens, considering the context on both sides, enabling it to capture detailed contextual information. VLMs like UNITER (Chen et al., 2020b) and VisualBERT (Li et al., 2019) utilize MLM during pre-training.

**Masked image modeling (MIM)**, extending the idea of MLM to images, emerged as a novel approach (Xie et al., 2022). In MIM, certain patches are masked, prompting the model to predict the contents of masked regions. This process enables the model to draw context from the entirety of the image, encouraging the integration of both local and global visual features. VLMs like UNITER (Chen et al., 2020b) and ViLBERT (Lu et al., 2019) leverage MIM for enhanced performance. The *cross-entropy loss* is employed in MLM and MIM tasks to measure the difference between predicted and actual probability distributions for the masked elements. Additionally, MLM can be combined with MIM, allowing the reconstruction of the masked signal in one modality with support from another modality (Kwon et al., 2023).

**Image-text matching (ITM)** is another common vision-language pre-training task. Throughout the training, the model learns to map images and corresponding textual descriptions into a shared semantic space, where closely aligned vectors represent similar content in both modalities. In single-stream VLMs, the special token [CLS] represents the joint representation for both modalities. In contrast, in dual-stream VLMs, the visual and textual representations of [CLS]_V_ and [CLS]_T_ are concatenated. This joint representation is fed into a fully-connected layer followed by the sigmoid function, predicting a score indicating match or mismatch (Chen et al., 2023). Models like CLIP (Radford et al., 2021) and ALBEF (ALign the image and text representations BEfore Fusing; Li et al., 2021) leverage ITM during pre-training.

In VLM pre-training, multiple tasks are often combined to enable models to understand nuanced contextual information across modalities. Tasks like contrastive loss, cross-entropy loss for masked token prediction, and others can be integrated into the final loss function. This approach equips VLMs with versatile representations for diverse downstream tasks. For instance, ALBEF (Li et al., 2021) adopts a pre-training objective involving CL, MLM, and ITM tasks, with the overall loss computed as the sum of these components.

#### 3.2.5 Fine-tuning techniques

Following the training, a common practice involves fine-tuning VLMs on smaller datasets tailored to specific downstream tasks. In the following, we present well-known techniques for fine-tuning VLMs.

**Supervised fine-tuning (SFT)** involves meticulous fine-tuning of a model on a dataset curated to match the nuances of the targeted application. However, before engaging in SFT, the VLM undergoes pre-training on an extensive image-text dataset to establish a foundational understanding of visual-textual relationships. This dual-phase strategy enables the model to generalize broadly while adapting to specific applications (Ouyang et al., 2022).

**Reinforcement learning from human feedback (RLHF)** is a distinct fine-tuning approach employed to enhance VLMs through the incorporation of human preferences during fine-tuning (Ouyang et al., 2022; Lambert et al., 2022; Ziegler et al., 2020). RLHF initiates with an initial model, incorporating human-generated rankings of its outputs to construct a detailed reward model. In contrast to traditional reinforcement learning (RL; Sutton and Barto, 1998; Coronato et al., 2020), which relies solely on environmental interactions, RLHF strategically integrates human feedback. This human-in-the-loop approach provides a more nuanced and expert-informed methodology, allowing for fine-tuning in alignment with human preferences, ultimately improving model outcomes.

**Instruction fine-tuning (IFT)** refers to refining a pre-trained language model by providing specific instructions or guidance tailored to a particular task or application (Ren et al., 2024). This process typically involves exposing the model to examples or prompts related to the desired instructions and updating its parameters based on the feedback received during this task-specific training phase. Medical VLM, RaDialog (Pellegrini et al., 2023), employs this fine-tuning technique.

### 3.3 Parameter-efficient fine-tuning

This section explores strategies for adapting VLMs while keeping the model's parameters frozen and only updating newly added layers. PEFT has emerged as a prominent approach, focusing on optimizing parameter utilization, especially in scenarios with limited labeled data for the target task. PEFT integrates task-specific parameters, called *adapters*, into a pre-trained model while retaining its original parameters. Adapter modules typically feature a bottleneck structure, projecting original features into a reduced dimension, applying non-linearity, and then projecting back to the original dimension. This design ensures parameter efficiency by minimizing the number of added parameters per task. Adapter modules, placed after each layer of the pre-trained model, capture task-specific details while preserving shared parameters, enabling seamless extension to new tasks without significant interference with previously acquired knowledge.

#### 3.3.1 Low-rank adaptation

LoRA is a common adapter-based method (Hu et al., 2022). The adaptation process involves fine-tuning two smaller low-rank matrices that are decompositions of the larger weight matrix of the pre-trained model. These smaller matrices constitute the LoRA adapter modules, and the approach focuses on making low-rank modifications to adapt the model for specific tasks efficiently. Pre-trained LLMs that are part of medical VLMs architecture are often fine-tuned using LoRA (e.g., Visual Med-Alpaca (Shu et al., 2023) and RaDialog (Pellegrini et al., 2023)).

#### 3.3.2 Prompt tuning

Prompt tuning involves creating continuous vector representations as input hints (Lester et al., 2021), enabling the model to dynamically create effective prompts during training. This iterative process significantly enhances the model's ability to generate contextually relevant responses and adapt its behavior based on an evolving task. VLMs like Qwen-VL and InstructBLIP used prompt tuning (Bai et al., 2023a; Dai et al., 2023).

#### 3.3.3 Prefix token tuning

Prefix token tuning adds task-specific vectors to the input, specifically to the initial tokens known as *prefix tokens*, to guide the model's behavior for a given task (Li and Liang, 2021). For instance, VL-T5 utilized different prefixes for questions from various datasets (Cho et al., 2021). These vectors can be trained and updated independently while the remaining pre-trained model parameters are frozen. Prefix token tuning allows task-specific adaptation without compromising the pre-trained knowledge encoded in most model parameters.

### 3.4 In-context learning

In this section, we explore strategies for adapting VLMs using the context only, keeping the model's parameters (and PEFT/LoRA adapters, if any) frozen. In our settings, in-context learning may be considered using LLMs or VLMs for inference only.

#### 3.4.1 Prompt engineering

Prompt engineering involves guiding a trained model with task-specific instructions, known as *prompts*, to tailor its output for specific tasks (Gu et al., 2023). Examples include instructing the model to generate a radiology report for a specific image (e.g., RAMM; Pellegrini et al., 2023). Prompt engineering can also expose the VLM to interconnected examples or prompts, guiding it to a desired output. Another approach incorporates progressively structured instructions or questions, refining focus and enhancing the model's ability to generate coherent and contextually relevant responses (Gu et al., 2023).

#### 3.4.2 Retrieval augmented generation

RAG is a form of prompt engineering that involves strategically crafting prompts for both retrieval and generation phases, allowing for an adaptive and efficient process that leverages external knowledge sources to enhance generative tasks. While the original concept of RAG was developed in the context of NLP (Lewis et al., 2020), the principles behind retrieval and generation can be extended to multimodal learning (Zhao et al., 2023), including VLMs. RAG has been used in medical VLMs for tasks like VQA (e.g., RAMM; Yuan et al., 2023) and RG (e.g., CXR-RePaiR-Gen; Ranjit et al., 2023). RAG begins with a retrieval component, usually a pre-trained model designed for information retrieval. This versatile component excels in extracting pertinent information from extensive datasets, catering to various modalities such as images, text, codes, video, or audio when presented with diverse inputs (Zhao et al., 2023). Following the retrieval phase, the model returns a set of contexts related to the given input. The second component is a generative LLM. This component takes the input and the retrieved context and generates the final output. The generated output is conditioned on the input and the information extracted from the retrieved context. An intrinsic advantage of RAG lies in its capacity to reduce the reliance on extensive labeled datasets. While the base model is typically frozen during RAG, there are instances, as seen in RAMM (Yuan et al., 2023), where model parameters are updated in the process.

### 3.5 Downstream tasks

Multimodal downstream tasks leverage the acquired knowledge from pre-training VLMs to excel in diverse applications that require a joint understanding of visual and textual data.

#### 3.5.1 Report generation

RG is a prominent example of a typical medical VLM task, which centers on creating a comprehensive summary report of visual data. RG plays a crucial role in automatically summarizing diagnostic imaging results and reducing the workload of report writing (Monshi et al., 2020; Ting et al., 2023; Mohsan et al., 2023). For instance, in radiology, a report generation system could analyze a set of medical images such as X-rays, CT scans, or MRIs and generate a detailed report summarizing the observed abnormalities, their locations, and potential implications for diagnosis or treatment (Liu et al., 2023b). A radiology report usually has several sections: (1) *Examination* (type of exam), (2) *Indication* (reasons for the examination), (3) *Comparison* (prior exams), (4) *Technique* (scanning method) (5) *Findings* (detailed observations made by a radiologist), and (6) *Impression* (summary of the major findings; Mabotuwana et al., 2020). In the context of RG, VLMs are usually designed to generate *Findings* and *Impression* sections (Thawkar et al., 2023).

Traditional methods of RG in radiology, such as handwriting, telephone dictation, transcriptionist-oriented systems, speech recognition, and structured data entry, face several challenges, including medical errors, cognitive overload, and inefficient decision-making. Handwriting and telephone dictation are particularly vulnerable to mistakes, as they can suffer from issues like illegible handwriting and miscommunication, leading to misinterpretations. Structured data entry, although designed to standardize and streamline reporting, often places a significant cognitive burden on radiologists, who must meticulously input detailed information, potentially leading to fatigue and errors. While technological advancements like electronic health records (EHRs), improved speech recognition software, standardized reporting templates, and automated error detection have been developed to mitigate these challenges, they have limitations. For example, EHRs and speech recognition still require substantial manual input and proofreading, which can be time-consuming and prone to error. Standardized reporting templates are helpful in ensuring consistency, but they can be inflexible and may not always capture the nuanced details of individual cases. Automated error detection systems are also not foolproof, often requiring human oversight to verify and correct flagged issues. Despite these improvements, the need for manual effort and the potential for human error remain significant concerns.

The evolution of RG methods parallels the advancements in image captioning. Early methods in image captioning included retrieval-based approaches, where captions were generated by retrieving existing phrases from a database, and template-based approaches, where predefined sentence templates were filled with identified image elements, such as objects, actions, or locations (Bai and An, 2018). However, these approaches struggled with generating captions for unseen images. This limitation motivated the emergence of DL methods for RG. Initial DL approaches utilized CNNs to extract visual features from images, which were then processed by RNNs to generate text descriptions (Ting et al., 2023). While this CNN-RNN approach improved the flexibility of captioning, it still faced challenges in capturing complex relationships between images and text outputs, and it struggled with generating longer, more comprehensive reports, often required in the medical field. These challenges gradually led to the adoption of VLMs in medical RG.

VLMs represent a transformative leap in medical RG by addressing the shortcomings of previous methods. By simultaneously integrating imaging and textual data, VLMs are able to generate more comprehensive and coherent reports. They also significantly reduce cognitive load by automating the creation of comprehensive reports, thereby liberating clinicians from the repetitive and time-consuming task of manual report writing. Furthermore, VLMs provide consistent interpretations of imaging data, which helps minimize the risk of errors associated with clinician fatigue or oversight. Their capability to process large volumes of data efficiently streamlines the reporting process, enhancing the overall effectiveness of medical practice and contributing to more accurate diagnoses. Currently, VLMs tailored for RG are predominantly utilized for radiology images, with lesser application in other medical imaging domains such as pathology (Sengupta and Brown, 2023), robotic surgery (Xu et al., 2021), and ophthalmology (Li et al., 2022).

#### 3.5.2 Visual question answering

VQA is another important visual-language understanding task, where the model needs to comprehend images or videos and the posed question to provide a relevant and accurate response (Antol et al., 2015). The spectrum of questions encountered in VQA is broad, encompassing inquiries about the presence of specific objects, their locations, or distinctive properties within the image. In the medical context (Lin et al., 2023b), this may involve questions regarding the presence of medical conditions or abnormalities, such as “What abnormality is seen in the image?” (Ionescu et al., 2021) or “Is there gastric fullness?” (Lau et al., 2018). Other queries may delve into details like the imaging method used (Abacha et al., 2019), the organ system involved (Lau et al., 2018), or the presence of specific anatomical structures (Liu et al., 2021a).

Questions in VQA fall into two categories. *Open-ended questions* elicit responses in the form of phrases or sentences, fostering detailed and nuanced answers (Thawkar et al., 2023). On the other hand, *closed-ended questions* are designed to prompt limited responses, often with predetermined options, such as a short list of multiple choices, a yes/no response, or a numeric rating (Bazi et al., 2023). The task of VQA is commonly approached as either a classification task, a generation task, or both (Lin et al., 2023b). In the classification approach, models select the correct answer from a predefined set, while in the generation task, models produce free-form textual responses unconstrained by predefined options.

#### 3.5.3 Other tasks

Beyond VQA and RG, a spectrum of VLM tasks exist for the vision-language understanding (Chen et al., 2023). For instance, *referring expression comprehension* entails a model locating the specific area or object in an image that the given phrase or sentence refers to (Zhang et al., 2018). *Visual commonsense reasoning* involves answering questions about an image, typically presented in a multiple-choice format, and justifying the answer based on the model's understanding of the image and common sense knowledge (Zellers et al., 2019). *Vision-language retrieval* focuses on either generating or retrieving relevant information from images using textual data, or vice versa, obtaining information from text using visual data (Zhen et al., 2019). In the context of *visual captioning*, the model's role is to generate a concise, text-based description of either an image (Sharma et al., 2023). It is worth highlighting that some of these tasks can seamlessly transition from images to videos, showcasing the adaptability and versatility of VLMs across diverse visual contexts (Gan et al., 2022).

## 4 Medical VLMs

### 4.1 Medical datasets for VLMs

The adaptation of VLMs to various medical tasks is achieved through their pre-training and fine-tuning using specialized task-specific datasets. Below is the list of vision-language datasets available in the public domain that contain medical image-text pairs or question-answer (QA) pairs. Most of them are employed by medical VLMs described in Section 4.3 for pre-training, fine-tuning, and evaluating VQA and RG tasks. The comparative analysis of these datasets is presented in Table 1. Note that determining which dataset is best suited for a particular task can be challenging, as each medical application presents its own nuances and requirements. Factors such as the context in which images are acquired and the types of annotations provided can significantly influence a dataset's effectiveness for specific tasks. In some cases, it may be necessary to enhance existing datasets by adding relevant image-text pairs or QA pairs, or even to create entirely new datasets tailored to specific research questions or clinical scenarios.

**Table 1:** A list of datasets used for developing medical VLMs.

**Dataset**	**# image-text pairs**	**# QA pairs**	**Other components**	**Link**
**ROCO** Pelka et al. (2018)	81, 825	–	–	GH
**MIMIC-CXR** Johnson et al. (2019a)	377, 110	–	–	PN
**MIMIC-CXR-JPG** Johnson et al. (2019b)	377, 110	–	pathology labels	PN
**MIMIC-NLE** Kayser et al. (2022)	38, 003	–	diagnosis labels, evidence labels	GH
**CXR-PRO** Ramesh et al. (2022)	–	–	374, 139 radiographs and 374, 139 reports but not paired	PN
**MS-CXR** Boecking et al. (2022)	1, 162	–	bounding box annotations	PN
**IU-Xray or Open-I** Demner-Fushman et al. (2015)	7, 470	–	labels	Web
**MedICaT** Subramanian et al. (2020)	224, 567	–	annotations; inline references to ROCO figures	GH
**PMC-OA** Lin et al. (2023a)	1, 650, 000	–	–	HF
**SLAKE** Liu et al. (2021a)	–	14, 028	642 annotated images, 5, 232 medical triplets	Web
**VQA-RAD** Lau et al. (2018)	–	3, 515	315 radiology images	Web
**PathVQA** He et al. (2020)	–	32, 799	4, 998 pathology images	GH
**VQA-Med 2019** Abacha et al. (2019)	–	15, 292	4, 200 radiology images	GH
**VQA-Med 2020** Abacha et al. (2020)	–	5, 000	5, 000 radiology images for VQA; images and questions for VQG	GH
**VQA-Med 2021** Ionescu et al. (2021)	–	5, 500	5, 500 radiology images for VQA; images and questions for VQG	GH
**EndoVis 2017** Allan et al. (2019)	–	472	bounding box annotations; 97 frames	GH
**EndoVis 2018** Allan et al. (2020)	–	11, 783	bounding box annotations; 2,007 frames	GH + Web
PathQABench-Public Lu et al. (2024b)	–	312	52 ROIs from WSIs	GH
GH, GitHub; HF, Hugging Face; PN, PhysioNet.				

Datasets with image-text pairs are typically employed for training medical VLMs, as well as for fine-tuning and evaluating models on RG tasks. Additionally, datasets containing question-answer (QA) pairs are specifically designed for fine-tuning and evaluating models in VQA tasks. GH - GitHub, HF - Hugging Face, and PN - PhysioNet.

#### 4.1.1 Radiology objects in context

ROCO is a dataset composed of image-caption pairs extracted from the open-access biomedical literature database PubMed Central (PMC; Pelka et al., 2018). ROCO is stratified into two categories: radiology and out-of-class. The radiology group includes 81, 825 radiology images, including CT, ultrasound, x-ray, fluoroscopy, positron emission tomography (PET), mammography, MRI, angiography, and PET-CT. The out-of-class group has 6, 127 images, including synthetic radiology images, clinical photos, portraits, compound radiology images, and digital art. To facilitate model training, the dataset is randomly split into a training set (65, 460 radiology and 4, 902 out-of-class images), a validation set (8, 183 radiology and 612 out-of-class images), and a test set (8, 182 radiology and 613 out-of-class images) using an 80/10/10 split ratio, respectively.

#### 4.1.2 Medical information mart for intensive care—chest X-ray

MIMIC-CXR collection encompasses 377, 110 chest X-rays paired with 227, 835 associated free-text radiology reports (Johnson et al., 2019a). The dataset is derived from de-identified radiographic studies conducted at the Beth Israel Deaconess Medical Center in Boston, MA. Each imaging study within the MIMIC-CXR dataset consists of one or more images, typically featuring lateral and from back-to-front (posteroanterior, PA) views in Digital Imaging and Communications in Medicine (DICOM) format.

#### 4.1.3 MIMIC-CXR-JPG

MIMIC-CXR-JPG (Johnson et al., 2019b) is a pre-processed variant of the MIMIC-CXR dataset (Johnson et al., 2019a). In this version, the original 377, 110 images are converted into compressed JPG format. The 227, 827 reports associated with these images are enriched with labels for various common pathologies. The labels are derived from the analysis of the impression, findings, or final sections of the radiology reports, facilitated by the use of NegBio (Peng et al., 2017) and CheXpert (Chest eXpert; Irvin et al., 2019) tools.

#### 4.1.4 MIMIC-NLE

MIMIC-NLE dataset is specifically designed for the task of generating natural language explanations (NLEs) to justify predictions made on medical images, particularly in the context of thoracic pathologies and chest X-ray findings Kayser et al. (2022). The dataset consists of 38, 003 image-NLE pairs or 44, 935 image-diagnosis-NLE triplets, acknowledging instances where a single NLE may explain multiple diagnoses. NLEs are extracted from MIMIC-CXR Johnson et al. (2019a) radiology reports. The dataset exclusively considers X-ray views from front-to-back (anteroposterior, AP) and back-to-front (posteroanterior, PA). All NLEs come with diagnosis and evidence (for a diagnosis) labels. The dataset is split into the training set with 37, 016 images, a test set with 273 images, and a validation set with 714 images.

#### 4.1.5 CXR with prior references omitted

CXR-PRO dataset is derived from MIMIC-CXR (Johnson et al., 2019a). The dataset consists of 374, 139 free-text radiology reports containing only the impression sections (Ramesh et al., 2022). It also incorporates associated chest radiographs; however, the radiology reports and chest X-rays are not paired. This dataset is designed to mitigate the problem of hallucinated references to prior reports often generated by radiology report generation ML models. The omission of prior references in this dataset aims to provide a cleaner and more reliable dataset for radiology RG.

#### 4.1.6 Indiana University chest X-rays

IU-Xray dataset, also known as the *Open-I* dataset, is accessible through the National Library of Medicine's Open-i service (Demner-Fushman et al., 2015). The dataset originates from two hospital systems within the Indiana Network for Patient Care database. This dataset comprises 7, 470 DICOM chest X-rays paired with 3, 955 associated radiology reports. Indication, finding, and impression sections are manually annotated using MeSH and RadLex (Radiology Lexicon) codes to represent clinical findings and diagnoses. Throughout this review, we will refer to the dataset interchangeably as *IU-Xray* and *Open-I*, maintaining consistency with the nomenclature used in related literature.

#### 4.1.7 Medical images, captions, and textual references

MedICaT dataset contains 217, 060 figures from 131, 410 open-access PMC papers focused on radiology images and other medical imagery types (Subramanian et al., 2020). Excluding figures from ROCO (Pelka et al., 2018), the dataset integrates inline references from the S2ORC (Semantic Scholar Open Research Corpus; Lo et al., 2020) corpus, establishing connections between references and corresponding figures. Additionally, the inline references to ROCO figures are provided separately. MedICaT also contains 7, 507 subcaption-subfigure pairs with annotations derived from 2, 069 compound figures.

#### 4.1.8 PubMedCentral's OpenAccess

PMC-OA dataset comprises 1.65 M image-caption pairs, derived from PMC papers (Lin et al., 2023a). It encompasses a variety of diagnostic procedures, including common ones such as ultrasound, MRI, PET, and radioisotope, and rarer procedures like mitotic and fMRI. Additionally, the dataset covers a broad spectrum of diseases, with induced cataracts, ear diseases, and low vision being among the most frequently represented conditions.

#### 4.1.9 MS-CXR

MS-CXR dataset contains image bounding box labels paired with radiology findings, annotated and verified by two board-certified radiologists (Boecking et al., 2022). The dataset consists of 1, 162 image-text pairs of bounding boxes and corresponding text descriptions. The annotations cover 8 different cardiopulmonary radiological findings and are extracted from MIMIC-CXR (Johnson et al., 2019a) and REFLACX (Reports and Eye-tracking data For Localization of Abnormalities in Chest X-rays; Bigolin Lanfredi et al., 2022; based on MIMIC-CXR) datasets. The findings include atelectasis, cardiomegaly, consolidation, edema, lung opacity, pleural effusion, pneumonia, and pneumothorax.

#### 4.1.10 Semantically-labeled knowledge-enhanced

SLAKE is an English-Chinese bilingual dataset (Liu et al., 2021a). It contains 642 images, including 12 diseases and 39 organs of the whole body. Each image is annotated with two types of visual information: masks for semantic segmentation and bounding boxes for object detection. The dataset includes a total of 14, 028 QA pairs, categorized into vision-only or knowledge-based types and labeled accordingly, encompassing both open- and closed-ended questions. Moreover, SLAKE incorporates 5, 232 medical knowledge triplets in the form of < *head, relation, tail*>, where *head* and *tail* denote entities (e.g., organ, disease), and *relation* signifies the relationship between these entities (e.g., function, treatment). An illustrative example of such a triplet is < pneumonia, location, lung>.

#### 4.1.11 VQA-RAD

VQA-RAD dataset contains 104 head axial single-slice CTs or MRIs, 107 chest x-rays, and 104 abdominal axial CTs (Lau et al., 2018). The images are meticulously chosen from MedPix, an open-access online medical image database, ensuring each image corresponds to a unique patient. Furthermore, every selected image has an associated caption and is deliberately devoid of any radiology markings. Every caption provides details about the imaging plane, modality, and findings generated and reviewed by expert radiologists. Also, VQA-RAD contains 3, 515 QA pairs, with an average of 10 questions per image. Among them, 1, 515 are free-form questions and answers, allowing for unrestricted inquiry. Additionally, 733 pairs involve rephrased questions and answers, introducing linguistic diversity. Another 1, 267 pairs are framed, featuring questions presented in a structured format, offering consistency and systematic evaluation. Additionally, QA pairs are split into 637 open-ended and 878 closed-ended types. Within the closed-ended group, a predominant focus is on yes/no questions.

#### 4.1.12 PathVQA

PathVQA is a dataset that encompasses 4, 998 pathology images accompanied by a total of 32, 799 QA pairs derived from these images (He et al., 2020). The images are sourced from pathology books: “Textbook of Pathology” and “Basic Pathology,” and the digital library “Pathology Education Informational Resource”. Out of all QA pairs, 16, 465 are of the open-ended type, while the remaining pairs are of the closed-ended yes/no type. On average, each image is associated with 6.6 questions, which cover a broad spectrum of visual contents, encompassing aspects such as color, location, appearance, shape, etc.

#### 4.1.13 VQA-Med 2019

VQA-Med 2019 dataset contains 4, 200 radiology images obtained from MedPix, an open-access online medical image database, and 15, 292 QA pairs (Abacha et al., 2019). The training set consists of 3, 200 images and 12, 792 QA pairs, with each image having 3 to 4 associated questions. The validation set includes 500 images and 2, 000 QA pairs, and the test set comprises 500 images and 500 QA pairs. The questions are mainly about modality, imaging plane, organ system, and abnormality.

#### 4.1.14 VQA-Med 2020

VQA-Med 2020 dataset contains 5, 000 radiology images obtained from MedPix, an open-access online medical image database, and 5, 000 QA pairs (Abacha et al., 2020). The training set consists of 4, 000 images and 4, 000 QA pairs. The validation set comprises 500 images and 500 QA pairs, and the test set includes 500 images and 500 QA pairs. The questions are focused on abnormalities present in the images. Additionally, the dataset contains radiology images and questions for the Visual Question Generation (VQG) task. The training set consists of 780 images and 2, 156 associated questions. The validation set comprises 141 images with 164 questions, and the test set includes 80 images.

#### 4.1.15 VQA-Med 2021

VQA-Med 2021 dataset contains 5, 500 radiology images obtained from MedPix, an open-access online medical image database, and 5, 500 QA pairs (Ionescu et al., 2021). The training set consists of 4, 500 images and 4, 5000 QA pairs. The validation set comprises 500 images and 500 QA pairs, and the test set includes 500 images and 500 QA pairs. The questions are focused on abnormalities present in the images. Similarly to VQA-Med 2019, the dataset also contains radiology images and questions for the VQG task. The validation set comprises 85 images with 200 questions, and the test set includes 100 images.

#### 4.1.16 Endoscopic vision 2017

EndoVis 2017 dataset contains 5 robotic surgery videos (two videos with 8 frames each, one with 18, one with 14, and one with 39 frames) from the MICCAI (Medical Image Computing and Computer Assisted Interventions) Endoscopic Vision 2017 Challenge (Allan et al., 2019). It also includes 472 QA pairs with bounding box annotations. These QA pairs are carefully crafted to involve specific inquiries related to the surgical procedure. Examples of questions include queries such as “What is the state of prograsp forceps?” and “Where is the large needle driver located?” The inclusion of bounding box annotations enhances the dataset's utility for tasks such as object detection or answer localization.

#### 4.1.17 EndoVis 2018

EndoVis 2018 dataset contains 14 robotic surgery videos (2, 007 frames in total) from the MICCAI Endoscopic Vision 2018 Challenge (Allan et al., 2020). It also includes 11, 783 QA pairs regarding organs, surgical tools, and organ-tool interactions. When the question is about organ-tool interactions, the bounding box will contain both the organ and the tool.

#### 4.1.18 PathQABench-Public

PathQABench-Public contains 52 regions of interest (ROIs) hand-selected by a board-certified pathologist from whole slide images (WSIs) in the publicly available The Cancer Genome Atlas (TCGA) repository. These images represent various organ systems: brain, lung, gastrointestinal tract, urinary tract, male reproductive tract, skin/eye/connective tissue, pancreaticohepatobiliary system, endocrine system, head/neck/mediastinum, gynecology, and breast. Per each organ system there are from 4 to 6 images. Each image is paired with a corresponding multiple-choice question, offering 10 possible answers. Additionally, there are five open-ended questions for each image, resulting in a total of 260 open-ended questions categorized into microscopy, diagnosis, clinical, and ancillary testing.

### 4.2 VLM evaluation metrics

This section delves into the evaluation process of medical VLMs. The initiation of this process involves meticulously selecting benchmark datasets and defining evaluation metrics tailored to the specific vision-language tasks at hand.

#### 4.2.1 Evaluation metrics for report generation

The prevalent benchmark datasets for medical RG are MIMIC-CXR (Johnson et al., 2019a) and Open-I (Demner-Fushman et al., 2015). For more information on these datasets, see Section 4.1. Several metrics are used to evaluate the effectiveness of VLMs on RG tasks. The more frequently used metrics are outlined below.

**Bilingual Evaluation Understudy (BLEU)** score was originally designed for machine translation evaluation, but it has been adapted for RG and even VQA in a modified form. BLEU provides a quantitative measure of how well the machine-generated text aligns with human-generated reference text (Papineni et al., 2002). First, the precision of different *n-grams*, which are consecutive sequences of *n* words, is calculated using the formula:


(1) $$\begin{array}{l} \text{Precision}\left(n\right)=\frac{\#\text{overlapping n-grams}}{\#\text{all n-grams in a model-generated text}}, \end{array}$$

where “overlapping n-grams” refer to n-grams in the model-generated text that share common elements with at least one n-gram in the reference text. To ensure the precision score remains robust and is not disproportionately affected by repeated n-grams in the model-generated text, a modification known as clipping is often introduced. This process involves capping the count of each n-gram in the model-generated text to a maximum count. This maximum count is determined by the highest count observed in any single reference text for the same n-gram. The final BLEU-n score is defined as:


(2) $$\begin{array}{l} \text{BLEU-n}=BP\times \frac{1}{n}\exp\left(\overset{n}{\underset{k=1}{\sum }}\log\left[\text{Precision(k)}\right]\right). \end{array}$$

In eq. 2, *BP* is referred to as the brevity penalty and is calculated as:


(3) $$\begin{array}{l} BP=\{\begin{array}{ll} 1 & \text{if }c\geq r\\ e^{\left(1-r/c\right)} & \text{if }c<r, \end{array} \end{array}$$

where *c* is the length of the model-generated text, and *r* is the length of the reference text. It is common to use *n* = 4. The BLEU score ranges from 0 to 1, where a higher score suggests better agreement with the reference text. The overall BLEU score of the model is the average of BLEU scores for each pair of reports.

**Recall-Oriented Understudy for Gisting Evaluation (ROUGE)** is a set of metrics that evaluate the overlap between the model-generated text and human-generated reference text (Lin, 2004). ROUGE-n assesses the overlap of n-grams between model-generated text and reference text, and it is defined as:


(4) $$\begin{array}{l} \text{ROUGE-n}=\frac{\#\text{overlapping n-grams}}{\#\text{all n-grams in a reference text}}. \end{array}$$

ROUGE-L focuses on measuring the longest common subsequence between model-generated text *Y* and reference text *X*, and it is calculated using the following relationship:


(5) $$\begin{array}{l} \text{ROUGE-L}=\frac{\left(1+\beta^{2}\right)\times R\times P}{\left(R+P\times \beta^{2}\right)}, \end{array}$$

where *R* = *LCS*(*X, Y*)/*m*, *P* = *LCS*(*X, Y*)/*n*, *m* is the length of *X*, *n* is the length of *Y*, *LCS*(*X, Y*) is the length of a longest common subsequence of *X* and *Y*, and β is a parameter that depends on the specific task and the relative importance of precision (P) and recall (R). There are other ROUGE score variants. The ROUGE scores range from 0 to 1, where higher scores indicate similarity between the model-generated text and the reference text. For each ROUGE variant, the overall score of the model is the average of scores for each instance.

**Metric for Evaluation of Translation with Explicit ORrdering (METEOR)** is an evaluation metric designed to be more forgiving than some other metrics and takes into account the fluency and meaning of the generated text (Banerjee and Lavie, 2005). The METEOR score is computed as follows:


(6) $$\begin{array}{l} \text{METEOR}=\frac{10\times P\times R}{R+9\times P}\left(1-\text{Penalty}\right) \end{array}$$

where


(7) $$\begin{array}{l} R=\frac{\#\text{overlapping 1-grams}}{\#\text{1-grams in a reference text}}, \end{array}$$

(8) $$\begin{array}{l} P=\frac{\#\text{overlapping 1-grams}}{\#\text{1-grams in a model-generated text}}, \end{array}$$

(9) $$\begin{array}{l} \text{Penalty}=\frac{1}{2}\times {\left(\frac{\#\text{chunks}}{\#\text{overlapping 1-grams}}\right)}^{3}, \end{array}$$

and *chunks* are groups of adjacent 1-grams in the model-generated text that overlap with adjacent 1-grams in the reference text. The METEOR score ranges from 0 to 1, with higher scores indicating better alignment between the model-generated text and the reference text. The overall METEOR score of a model is the average of scores for each instance.

**Perplexity** measures the average uncertainty of a model in predicting each word in a text (Hao et al., 2020). The formula for perplexity is defined as:


(10) $$\begin{array}{l} \text{Perplexity}=\exp\left(-\frac{1}{n}\overset{n}{\underset{k=1}{\sum }}\ln\;P\left(w_{k}|w_{1},w_{2},\ldots ,w_{k-1}\right)\right), \end{array}$$

where *n* is the total number of words in the text. The value of the perplexity metric can range from 1 to +∞, and lower values signify a more accurate and confident model in capturing the language patterns within the given text.

**BERTScore** was initially designed for evaluating models that use BERT (Devlin et al., 2019) embeddings (Zhang et al., 2020). However, it can also leverage other word embeddings to evaluate the similarity between model-generated and reference text. The BERTScore of a single text pair is calculated according to the relationship:


(11) $$\begin{array}{l} \text{BERTScore}=\frac{2\times P\times R}{P+R}, \end{array}$$

where *P* represents the ratio of the maximum cosine similarity score between tokens in the model-generated text and the reference text to the numbers of tokens in the model-generated text and *R* represents the ratio of the maximum cosine similarity score between tokens in the model-generated text and the reference text to the numbers of tokens in the reference text. The BERTScore of the model is the average of BERTScores across all text pairs.

**RadGraph F1** is a novel metric that measures overlap in clinical entities and relations extracted from radiology reports (Yu et al., 2023). The RadGraph F1 score is computed in the following way. First, the RadGraph model maps model-generated and reference reports into graph representations with clinical entities represented as nodes and their relations as edges between them. Second, the number of nodes that match between the two graphs based on clinical entity text and labels (entity type) is determined. Third, the number of edges that match between the two graphs based on their start and end entities and labels (relation type) is calculated. Lastly, the F1 score is separately computed for clinical entities and relations, and then the RadGraph F1 score for a report pair is the average of these two scores. The overall model performance is determined by averaging RadGraph F1 scores across all report pairs.

**Human evaluation** is crucial for assessing the quality of VLMs in medical RG. In Jeong et al. (2023), expert radiologists assessed the X-REM model's performance in RG by segmenting reports into lines and assigning scores based on five error categories to each line. These scores reflected error severity, with higher values indicating more severe errors.

The next few metrics are designed for classification evaluation, and RG can be viewed as such a task. In Moon et al. (2022), Lee et al. (2023), and Pellegrini et al. (2023), these metrics are computed based on the 14 labels obtained from applying the CheXpert (Irvin et al., 2019) or CheXbert (Smit et al., 2020) labeler to the reference reports as well as the model-generated reports. In this context, reports bearing accurate diagnosis labels are categorized as positive, while those with inaccurate labels are regarded as negative. The following metrics are also called clinical efficacy metrics.

- *Accuracy* measures the ratio of all positive predictions to the total number of predictions.
- *Precision* evaluates the accuracy of positive predictions. It is calculated as the ratio of true positive predictions to the total instances predicted as positive, expressed as:

(12) $$\begin{array}{l} \text{Precision}=\frac{\text{True Positives}}{\text{True Positives }+\text{ False Positives}}. \end{array}$$

High Precision indicates a low false positive rate.

- *Recall* assesses the model's ability to predict all positive classes. It is defined as the ratio of correctly predicted positive observations to the total actual positives:

(13) $$\begin{array}{l} \text{Recall}=\frac{\text{True Positives}}{\text{True Positives }+\text{ False Negatives}}. \end{array}$$

High Recall means effectively identifying the most actual positive instances.

- *F1 Score* provides an overall measure of the model's performance by balancing Precision and Recall. It is calculated as:

(14) $$\begin{array}{l} \text{F1}=\frac{2\times \text{Precision}\times \text{Recall}}{\text{Precision }+\text{ False Recall}}. \end{array}$$

F1 scores range from 0 to 1, with higher values indicating better performance. In multi-class classification, the macro-F1 score is commonly computed by averaging the F1 scores independently calculated for each class. This method ensures unbiased evaluation across all classes, assigning equal importance regardless of size or prevalence.

#### 4.2.2 Evaluation metrics for VQA

The common benchmark datasets for medical VQA include VQA-RAD (Lau et al., 2018), SLAKE (Liu et al., 2021a), and PathVQA (He et al., 2020). While various metrics are available for VQA evaluation, only a few are highlighted here to avoid redundancy with already mentioned metrics.

**Accuracy** is a fundamental metric for gauging overall model correctness in VQA evaluation. It is determined by calculating the proportion of correctly predicted answers to the total number of questions. For a detailed comparison of accuracies among different medical VLMs discussed in Section 4.3, refer to Table 3.

**Exact match** computes the ratio of generated answers that match exactly (excluding punctuation) the correct answer. However, it may not credit semantically correct answers that lack an exact lexical match. This metric is more suitable for evaluating answers to close-ended questions than open-ended ones.

**Human evaluation** can be performed for VQA in various ways. For instance, in Moor et al. (2023), medical experts evaluated Med-Flamingo's performance on each VQA problem using a user-friendly interface, assigning scores from 0 to 10.

### 4.3 Medical models

In this part of the review paper, we provide an overview of existing medical VLMs tailored for VQA and/or RG. The information is organized chronologically based on the first appearance of the model. Our focus is mainly on recently introduced open-source and publicly available models. A summary of these VLMs is presented in Table 2.

**Table 2:** A list of medical VLMs developed for VQA and RG.

**Model**	**Stream**	**Decoder**	**Architecture**	**VQA**	**RG**	**Datasets**	**Code**
**MedViLL** Moon et al. (2022)	single	No	RN50 + BERT	+	+	MIMIC-CXR, Open-I, VQA-RAD	GH
**PubMedCLIP** Eslami et al. (2023)	dual	No	ViT-B/32 or RN50 or RN50 × 4 + Transformer + BAN	+	–	ROCO, SLAKE, VQA-RAD	GH
**RepsNet** Tanwani et al. (2022)	dual	Yes	ResNeXt-101 + BERT + BAN + language decoder	+	+	VQA-RAD, IU-Xray	Web
**BiomedCLIP** Zhang et al. (2023a)	dual	No	ViT-B/16 + PubMedBERT + METER	+	–	PMC-15, SLAKE, VQA-RAD	HF
**UniXGen** Lee et al. (2023)	single	Yes	VQGAN + Transformer	–	+	MIMIC-CXR	GH
**RAMM** Yuan et al. (2023)	dual	No	Swiss Transformer + PubMedBERT + multimodal encoder w/ retrieval-atten. module	+	–	PMCPM, ROCO MIMIC-CXR, SLAKE, VQA-RAD, VQA-Med 2019, VQA-Med 2021	GH
**X-REM** Jeong et al. (2023)	dual	No	ALBEF (ViT-B/16 + BERT + multimodal encoder)	–	+	MIMIC-CXR,MedNLI, RadNLI	GH
**Visual Med-Alpaca** Shu et al. (2023)	single	Yes	DePlot or Med-GIT + prompt manager +LLaMa-7B	+	–	ROCO; MedDialog, MEDIQA QA, MEDIQA RQE, MedQA, PubMedQA + GPT-3.5-Turbo	GH
**CXR-RePaiR-Gen** Ranjit et al. (2023)	dual	Yes	ALBEF + FAISS retriever + prompt manager + text-davinci-003 or GPT-3.5-Turbo or GPT-4	–	+	CXR-PRO, MS-CXR	–
**LLaVa-Med** Li et al. (2023a)	single	Yes	ViT-L/14 + projection layer + LLaMa-7B	+	–	PMC-15 + GPT-4, VQA-RAD, SLAKE, PathVQA	GH
**XrayGPT** Thawkar et al. (2023)	single	Yes	MedCLIP + linear transformation layer+ Vicuna-7B	+	+	MIMIC-CXR Open-I	GH
**CAT-ViL DeiT** Bai et al. (2023b)	dual	No	RN18 + CAT-ViL fusion module + DeiT	+	–	EndoVis 2017, EndoVis 2018	GH
**MUMC** Li et al. (2023b)	dual	Yes	ViT-B/12 + BERT + multimodal encoder + answer decoder	+	–	ROCO, MedICaT, ImageCLEF Caption, VQA-RAD, SLAKE PathVQA	GH
**Med-Flamingo** Moor et al. (2023)	single	Yes	ViT-L/14 + perceiver resampler + LLaMa-7B	+	–	MTB, PMC-OA, VQA-RAD, PathVQA,Visual USMLE	GH
**RaDialog** Pellegrini et al. (2023)	single	Yes	BioViL-T + BERT + prompt manager + Vicuna-7B	+	+	MIMIC-CXR,Instruct	GH
**PathChat** Lu et al. (2024b)	single	Yes	UNI + multimodal projector + Llama 2-13B	+	–	CONCH, PathChat dataset, PathQABench	GH

#### 4.3.1 Medical vision language learner

MedViLL can process medical images to generate associated reports (Moon et al., 2022). The model employs ResNet-50 (He et al., 2016), trained on ImageNet (Deng et al., 2009), for extracting visual features *v*. The model leverages WordPiece (Wu et al., 2016) tokenizer to extract textual features *t* from clinical reports. Both visual and textual features incorporate positional information to capture spatial relationships and sequential order. These features, along with special tokens [CLS], [SEP]_V_, [SEP]_L_, are concatenated into a single vector (*CLS, v, SEP*_*V*_, *t, SEP*_*L*_) and fed into the BERT-based Transformer. The MedViLL is pre-training on two tasks: MLM and ITM. The MLM task employs a bidirectional auto-regressive (BAR) self-attention mask. For MLM, a negative log-likelihood loss function is used. Pre-training is performed on 89, 395 image-report pairs from MIMIC-CXR (Johnson et al., 2019a), with fine-tuning on 3, 547 pairs from Open-I (Demner-Fushman et al., 2015). VQA is performed on VQA-RAD (Lau et al., 2018) (see Table 3), where the output representation of [CLS] is used to predict a one-hot encoded answer. For radiology RG fine-tuning, the model uses a sequence-to-sequence (S2S) mask instead of BAR and generates reports by sequentially recovering MASK tokens. RG is evaluated on MIMIC-CXR (Johnson et al., 2019a) and Open-I (Demner-Fushman et al., 2015). MedViLL achieves a BLEU-4 score of 0.066, a perplexity value of 4.185, and using a CheXpert labeler (Irvin et al., 2019) an accuracy of 84.1%, a precision value of 0.698, a recall value of 0.559, and an F1 score of 0.621 on MIMIC-CXR. Additionally, it achieves a BLEU-4 score of 0.049, a perplexity value of 5.637, an accuracy of 73.4%, a precision value of 0.512, a recall value of 0.594, and an F1 score of 0.550 on Open-I.

**Table 3:** The comparison of medical VLMs' accuracies on VQA tasks.

**Model**	**SLAKE**	**SLAKE**	**VQA-RAD**	**VQA-RAD**	**PathVQA**	**PathVQA**		
	**open -ended**	**close-ended**	**open -ended**	**close-ended**	**open-ended**	**close -ended**	**VQA-Med 2019**	**VQA-Med 2021**
**MedViLL** Moon et al. (2022)	–	–	59.50%	77.70%	–	–	–	–
**PubMedCLIP** Eslami et al. (2023)	78.40%	82.50%	60.10%	80.00%	–	–	–	–
**RepsNet** Tanwani et al. (2022)	–	–	–	87.05%	–	–	–	–
**BioMedCLIP** Zhang et al. (2023a)	82.50%	89.70%	67.60%	79.80%	–	–	–	–
**RAMM** Yuan et al. (2023)	82.48%	91.59%	67.60%	85.29%	–	–	82.13%	39.20%
**LLaVa-Med** Li et al. (2023a)	–	84.19%	–	85.34%	–	91.21%	–	–
**MUMC** Li et al. (2023b)	–	–	71.50%	84.20%	39.00%	90.4%	–	–

The underlined accuracies are the highest for a specific dataset.

#### 4.3.2 PubMedCLIP

PubMedCLIP is a CLIP-based (Radford et al., 2021) model pre-trained on the ROCO (Pelka et al., 2018) dataset (Eslami et al., 2023). It employs a CLIP text encoder based on the Transformer architecture and three distinct visual encoders: ViT-B/32 (Dosovitskiy et al., 2021), ResNet-50, and ResNet-50 × 4 (He et al., 2016). Following CLIP's approach, the model generates joint representations by computing cosine similarity between textual and visual features. The pre-training objective involves computing cross-entropy losses for vision and language, which are then averaged to derive an overall loss. Repurposed as a pre-trained visual encoder for VQA, PubMedCLIP's output is also concatenated with the output of a convolutional denoising autoencoder (CDAE) (Masci et al., 2011). Questions are encoded using GloVe (Pennington et al., 2014) word embeddings followed by an LSTM (Hochreiter and Schmidhuber, 1997). Image and question features are combined using *bilinear attention networks* (BAN; Kim et al., 2018), and the resulting representations are classified using a two-layer feedforward neural network. The VQA loss combines classification and image reconstruction losses. PubMedCLIP is fine-tuned on datasets like SLAKE (Liu et al., 2021a) and VQA-RAD (Lau et al., 2018). Its performance is compared with existing Medical VQA (MedVQA) methods, such as Mixture of Enhanced Visual Features (MEVF; Zhan et al., 2020) and question-conditioned reasoning (QCR; Liu et al., 2023a). PubMedCLIP, integrated into the QCR framework, achieves superior accuracies on VQA-RAD and SLAKE datasets compared to the MEVF framework. The highest accuracies of PubMedCLIP in the QCR framework on both datasets are shown in Table 3.

#### 4.3.3 RepsNet

RepsNet is designed for VQA tasks (Tanwani et al., 2022). It can generate automated medical reports and interpret medical images. The model employs a modified version of the pre-trained ResNeXt-101 (Xie et al., 2016) as its image encoder and utilizes pre-trained BERT (Devlin et al., 2019) as the text encoder, with text tokenization done through WordPiece (Wu et al., 2016). Fusion of image and question features is achieved using BAN (Kim et al., 2018). To align images with textual descriptions, the model employs bidirectional contrastive learning (Chen et al., 2020a). The language decoder, based on GPT-2, is adapted to incorporate image features and prior context, generating text sequences in an auto-regressive manner until an end-of-sequence token is produced. The overall loss function combines contrastive loss for encoding phase and cross-entropy loss for decoding phase. For VQA tasks, the model is fine-tuned and evaluated on VQA-RAD (Lau et al., 2018) (see Table 3). In contrast, for RG, fine-tuning and evaluation are done using IU-Xray (Demner-Fushman et al., 2015) dataset. On the IU-Xray dataset, RepsNet achieves BLEU-2, and BLEU-4 scores of 0.44 and 0.27, respectively.

#### 4.3.4 BiomedCLIP

BiomedCLIP is pre-trained on the specifically curated PMC-15 dataset that consists of 15 M figure-caption pairs derived from the PMC articles (Zhang et al., 2023a) but is not publicly available. The model architecture is similar to CLIP (Radford et al., 2021), except that the text encoder is a pre-trained PubMedBERT (Gu et al., 2021) model with WordPiece tokenizer (Wu et al., 2016). The model uses ViT-B/16 (Dosovitskiy et al., 2021) as the visual data encoder. For pre-training, the model adopts the CL approach, and to mitigate memory usage, it utilizes the sharding contrastive loss (Cherti et al., 2022). For adaptation to VQA, the model incorporates the METER (Dou et al., 2022) framework. This involves deploying a Transformer-based co-attention multimodal fusion module that produces cross-modal representations. These representations are then fed into a classifier for the final prediction of answers. The model is evaluated on VQA-RAD (Lau et al., 2018) and SLAKE (English; Liu et al., 2021a) datasets (see Table 3).

#### 4.3.5 Unified chest X-ray and report Generation model

UniXGen is a unified model that can generate both reports and view-specific X-rays (Lee et al., 2023). The model tokenizes chest X-rays leveraging VQGAN (Esser et al., 2021), a generative model that amalgamates generative adversarial networks (GANs) with vector quantization (VQ) techniques. VQGAN employs an encoder to transform input images into continuous representations, subsequently using vector quantization to discretize them into learnable codebook vectors. Additionally, VQGAN incorporates a decoder, translating these discrete codes back into images during the generation process. For chest X-rays, multiple views from the same study are tokenized into sequences of discrete visual tokens, demarcated by special tokens to distinguish perspectives. In the case of radiology reports, the model uses the byte-level BPE (Wang et al., 2020) tokenizer, augmented with sinusoid positional embedding for enhanced representation. The model is based on the Transformer architecture (Vaswani et al., 2017) with a multimodal causal attention mask, ensuring that each position in the sequence attends to all previous positions and not future ones. During training, multiple views of chest X-rays and a report embedding are concatenated randomly and fed into the Transformer. The model is optimized using the negative log-likelihood loss function. The model is trained on 208, 534 studies sampled from the MIMIC-CXR (Johnson et al., 2019a) dataset. Each study contains at most three chest X-rays representing PA (from back to front), AP (from front to back), and lateral views. On the MIMIC-CXR dataset, UniXGen achieves a BLEU-4 score of 0.050 and, using the CheXpert labeler (Irvin et al., 2019), attains a precision score of 0.431, a recall value of 0.410, and an F1 score of 0.420.

#### 4.3.6 Retrieval-augmented bioMedical multi-modal pretrain-and-finetune paradigm

RAMM, a retrieval-augmented VLM designed for biomedical VQA, integrates Swin Transformer (Liu et al., 2021b) as its image encoder and PubMedBERT (Gu et al., 2021) as its text encoder (Yuan et al., 2023). The visual and textual features are then fused by the multimodal encoder, a 6-layer Transformer (Vaswani et al., 2017). The model is pre-trained on the MIMIC-CXR (Johnson et al., 2019a) and ROCO (Pelka et al., 2018) datasets along with a newly curated PMC-Patients-Multi-modal (PMCPM) dataset, consisting of 398, 000 image-text pairs sampled from PMC-OA (Lin et al., 2023a) dataset. The pre-training objective function of RAMM is the sum of three tasks: CL, ITM, and MLM. Using CL, the model aligns images and texts using the cosine similarity metric. The VQA task is viewed as a classification problem, and the model is optimized using the cross-entropy loss function. During model fine-tuning, the retrieval-attention module fuses the representations of the image-question input with four representations of the retrieved image-text pairs from the pre-trained datasets. This lets RAMM to focus on relevant parts of the retrieved information when generating answers. The model is evaluated on VQA-Med 2019 (Abacha et al., 2019), VQA-Med 2021 (Ionescu et al., 2021), VQA-RAD (Lau et al., 2018), and SLAKE (Liu et al., 2021a) datasets (see Table 3).

#### 4.3.7 Contrastive X-ray REport match

X-REM is a retrieval-based radiology RG model that uses an ITM score to measure the similarity of a chest X-ray image and radiology report for report retrieval (Jeong et al., 2023). The VLM backbone of the model is ALBEF (Li et al., 2021). ALBEF utilizes ViT-B/16 (Dosovitskiy et al., 2021) as its image encoder and initializes the text encoder with the first 6 layers of the BERT (Devlin et al., 2019) base model. The multimodal encoder in ALBEF, responsible for combining visual and textual features to generate ITM scores, is initialized using the final six layers of the BERT base model. X-REM leverages ALBEF's pre-trained weights and performs further pre-training on X-rays paired with extracted impression sections (2, 192 pairs), findings sections (1, 597 pairs), or both (2, 192 pairs) from the MIMIC-CXR (Johnson et al., 2019a) dataset. Subsequently, the model is fine-tuned on the ITM task, where the scoring mechanism involves using the logit value for the positive class as the similarity score for image-text pairs. To address the positive skewness in medical datasets, 14 clinical labels obtained from the CheXbert (Smit et al., 2020) labeler are utilized. The model efficiently manages the computational burden associated with ITM scores by employing ALBEF's pre-aligned unimodal embeddings. This involves narrowing down the candidate reports based on high cosine similarity with the input image before computing ITM scores. Additionally, the text encoder undergoes fine-tuning on natural language inference (NLI) task, utilizing datasets such as MedNLI (Romanov and Shivade, 2018) and RadNLI Miura et al. (2021). This step is crucial for preventing the retrieval of multiple reports with overlapping or conflicting information. X-REM achieves a BLEU-2 score of 0.186 on the MIMIC-CXR (Findings only) dataset. The BERTScore of the model is 0.386 on MIMIC-CXR (Findings only) and 0.287 on MIMIC-CXR (Impressions and Findings).

#### 4.3.8 Visual Med-Alpaca

Visual Med-Alpaca is a biomedical FM designed for addressing multimodal biomedical tasks like VQA (Shu et al., 2023). The model processes image inputs through a classifier to select the appropriate module for converting visual information into text, with supported modules including DePlot (Liu et al., 2022) for plots and Med-GIT (Wang et al., 2022a) fine-tuned on the ROCO (Pelka et al., 2018) dataset for radiology images. The prompt manager combines textual information from images and text inputs to form prompts for the LLaMA-7B (Touvron et al., 2023a) model. However, before generating responses, LLaMa-7B undergoes both standard and LoRA (Hu et al., 2022) fine-tuning on a carefully curated set of 54, 000 medical QA pairs. The questions within this set are derived from question-answering datasets such as MEDIQA QA (Ben Abacha et al., 2019), MEDIQA RQE (Ben Abacha et al., 2019), MedQA (Jin et al., 2021), MedDialog (Zeng et al., 2020), and PubMedQA (Jin et al., 2019), with their corresponding answers synthesized using GPT-3.5-Turbo in the *self-instruct* (Wang et al., 2023b) manner. Human experts filter and edit the obtained QA pairs for quality and relevance. The evaluation of this model is still ongoing (Shu et al., 2023).

#### 4.3.9 Contrastive X-ray-report pair retrieval based generation

CXR-RePaiR-Gen, designed for radiology RG, integrates the RAG framework to address hallucinated references (Ranjit et al., 2023). The model leverages the pre-trained ALBEF (Li et al., 2021) previously utilized in CXR-ReDonE (Ramesh et al., 2022). Textual features are indexed in a vector database, Facebook AI Similarity Search (FAISS). When given a radiology image input, embeddings from the reports or sentences corpus with the highest dot-product similarity to the image embedding are retrieved. The CXR-PRO (Ramesh et al., 2022) dataset is employed for text retrieval to gather relevant impressions for generating the radiology report. The retrieved impression sections from the CXR-PRO dataset serve as the context for the prompt to an LLM, along with instructions to generate the radiology report. Two prompts are employed: one for the text-davinci-003 model and another for conversational RG with GPT-3.5-Turbo and GPT-4 models. The model is evaluated on MS-CXR (Boecking et al., 2022) and CXR-PRO datasets. A code has yet to be provided for this model. Evaluated on MS-CXR and CXR-PRO datasets, CXR-RePaiR-Gen achieves BERTScore scores of 0.2865 on CXR-PRO (GPT-4) and 0.1970 on MS-CXR (text-davinci-003). Its RadGraph F1 scores are 0.1061 on CXR-PRO (GPT-4) and 0.0617 on MS-CXR (text-davinci-003), employing three retrieval samples per input during RAG.

#### 4.3.10 Large language and vision assistant for biomedicine

LLaVa-Med, an adaptation of LLaVa (Liu et al., 2023c), is customized for the medical domain through training on instruction-following datasets (Li et al., 2023a). Visual features are extracted by the pre-trained CLIP visual encoder ViT-L/14 (Dosovitskiy et al., 2021), which can be substituted with BiomedCLIP (Zhang et al., 2023a). These features are mapped into textual embedding space via linear projection layer and combined with instructions before being input to the LLM LLaMa-7B (Touvron et al., 2023a), which can be replaced with Vicuna (Chiang et al., 2023). After initializing with the general-domain LLaVA, the model undergoes fine-tuning using curriculum learning. First, the model learns to connect visual elements in biomedical images to corresponding language descriptions, using a dataset of 600, 000 image-caption pairs from PMC-15, initially employed in BiomedCLIP. These image-caption pairs are transformed into an instruction-following dataset, where the instructions prompt the model to describe the corresponding image concisely or in detail. Given the language instruction and image input, the model is prompted to predict the original caption. The visual encoder and language model weights are frozen during this stage, with updates exclusively applied to the linear projection layer. The second stage of training focuses on aligning the model to follow diverse instructions. For this purpose, another instruction-following dataset is generated from PMC-15. Instructions for this dataset are designed to guide the GPT-4 model to generate multi-round questions and answers from the image caption and sentences from the original PMC paper mentioning the image (Li et al., 2023a). In this training phase, the model undergoes training on a set of 60, 000 images, each accompanied by its respective caption and multi-round questions and answers. Throughout this process, the weights of the visual encoder remain unchanged, preserving the previously acquired visual features. Meanwhile, the pre-trained weights of the projection layer and the language model undergo continuous updates. Lastly, for VQA, the model is fine-tuned and evaluated on VQA-RAD (Lau et al., 2018), SLAKE (Liu et al., 2021a), and PathVQA (He et al., 2020) (see Table 3).

#### 4.3.11 XrayGPT

XrayGPT is a conversational medical VLM specifically developed for analyzing chest radiographs (Thawkar et al., 2023). The VLM uses MedCLIP (Wang et al., 2022b) to generate visual features. These features undergo a meticulous transformation process: initially, they are mapped to a lower-dimensional space through a linear projection head and subsequently translated into tokens via a linear transformation layer. The model incorporates two text queries: an assistant query framing its purpose and a doctor's query guiding relevant information provision. Tokens generated from a visual input are concatenated with the tokenized queries and then fed into Vicuna-7B (Chiang et al., 2023), fine-tuned on 100, 000 patient-doctor and 20, 000 radiology conversations sourced from: ShareGPT.com. During training, the weights of the vision encoder and LLM are frozen while the weights of the linear transformation layer undergo updates. The model is first trained on 213, 514 image-text pairs from pre-processed MIMIC-CXR (Johnson et al., 2019a) dataset and then on 3, 000 image-text pairs from Open-I (Demner-Fushman et al., 2015) dataset. XrayGPT achieves ROUGE-1 = 0.3213, ROUGE-2 = 0.0912, and ROUGE-L = 0.1997 on MIMIC-CXR dataset.

#### 4.3.12 Co-attention gaTed vision-language data-efficient image transformer

CAT-ViL DeiT is a specialized VLM tailored for VQA within surgical scenarios, focusing on answer localization (Bai et al., 2023b). It integrates ResNet-18 (He et al., 2016) pre-trained on ImageNet (Deng et al., 2009) to generate visual features and custom BERT tokenizer (Seenivasan et al., 2022) for text encoding. The *Co-Attention gaTed Vision-Language* (CAT-ViL) module facilitates interaction between visual and textual features, fused via gating mechanisms to optimize multimodal embeddings. These embeddings are further processed by a pre-trained *Data-efficient image Transformer* (DeiT) module for optimal joint representation. For VQA, the model adopts a standard classification head, while for answer localization within images, it employs the *detection with transformers* (DETR; Carion et al., 2020) head. The overall loss function comprises cross-entropy as the classification loss and L1-norm, along with the *generalized intersection over union* (GIoU; Rezatofighi et al., 2019), serving as the localization loss. The model is trained on 1, 560 frames, and 9, 014 QA pairs from the surgical datasets EndoVis 2018 (Allan et al., 2020). The model achieved an accuracy of 61.92% on the remaining data from EndoVis 2018 and 45.55% on EndoVis 2017 (Allan et al., 2019) dataset.

#### 4.3.13 Masked image and text modeling with unimodal and multimodal contrastive losses

MUMC utilizes a ViT-B/12 (Dosovitskiy et al., 2021) as its image encoder, the first 6 layers of BERT (Devlin et al., 2019) as its text encoder, and the last 6 layers of BERT as its multimodal encoder (Li et al., 2023b). The multimodal encoder incorporates cross-attention layers to align visual and textual features. For pre-training, the model employs CL, MLM, and ITM. Also, the model utilizes a newly introduced *masked image strategy*, randomly masking 25% of image patches as a data augmentation technique. This exposes the model to a greater variety of visual contexts and enables learning representations that are more robust to partially occluded inputs. The pre-training is performed on ROCO (Radford et al., 2021), MedICaT (Subramanian et al., 2020), and Image Retrieval in Cross-Language Evaluation Forum (ImageCLEF) caption (Rückert et al., 2022) datasets. For VQA tasks, an answering decoder is added to generate answer text tokens. The encoder weights are initialized from pre-training, and the model is fine-tuned and evaluated on VQA-RAD (Lau et al., 2018), SLAKE (Liu et al., 2021a), and PathVQA (He et al., 2020) (see Table 3).

#### 4.3.14 Med-Flamingo

Med-Flamingo is a multimodal few-shot learner model based on the Flamingo (Alayrac et al., 2022) architecture, adapted to the medical domain (Moor et al., 2023). The model is pre-trained on the MTB (Moor et al., 2023) dataset, a newly curated collection comprising 4, 721 segments from various Medical TextBooks, encompassing textual content and images. Each segment is designed to contain at least one image and up to 10 images, with a specified maximum length. Also, it is pre-trained on 1.3 M image-caption pairs from the PMC-OA (Lin et al., 2023a) dataset. The model's few-shot capabilities are achieved through training on these mixed text and image datasets, enabling it to generalize and perform diverse multimodal tasks with only a few examples. The model utilizes a pre-trained frozen CLIP vision encoder ViT-L/14 for visual feature generation. To convert these visual features into a fixed number of tokens, the model employs a module known as the *perceiver resampler*, which is trained from scratch. Subsequently, these tokens and tokenized text inputs undergo further processing in a pre-trained frozen LLM LLaMA-7B (Touvron et al., 2023a), enhanced with gated cross-attention layers, which are trained from scratch. This augmentation aids in learning novel relationships and enhances training stability. Med-Flamingo's performance is evaluated on VQA-RAD (Lau et al., 2018) and PathVQA (He et al., 2020). The exact match scores for MedFlamingo demonstrate a few-shot performance of 0.200 on VQA-RAD and 0.303 on PathVQA. In contrast, the zero-shot performance yields an exact match score of 0.000 on VQA-RAD and 0.120 on PathVQA. Additionally, it is evaluated on a specifically created Visual United States Medical Licensing Examination (USMLE) dataset, comprising 618 challenging open-ended USMLE-style questions augmented with images, case vignettes, and tables of laboratory measurements, covering a diverse range of medical specialties.

#### 4.3.15 RaDialog

RaDialog is a VLM that integrates automated radiology RG with conversational assistance (Pellegrini et al., 2023). The model incorporates BioViL-T (Bannur et al., 2023), a hybrid model that fuses the strengths of ResNet-50 (He et al., 2016) and Transformer (Vaswani et al., 2017) architectures. Pre-trained on radiology images and reports, BioViL-T generates patch-wise visual features. The extracted features undergo alignment through a BERT (Devlin et al., 2019) model, transforming them into a concise representation of 32 tokens. The model incorporates the CheXpert classifier to offer organized findings in medical images. These findings are generated based on labels obtained from the CheXbert (Smit et al., 2020) model. The classifier is trained independently using labels predicted by CheXbert from the findings section of radiology reports. Visual features, structured findings, and a directive prompt are combined as input for the Vicuna-7B LLM, fine-tuned using LoRA. The training is performed on MIMIC-CXR (Johnson et al., 2019a) dataset. RaDialog achieves a BLEU-4 score of 0.095, ROUGE-L score of 0.2710, METEOR score of 0.14, and BERTScore of 0.400 on the MIMIC-CXR dataset. To address the challenge of catastrophic forgetting during training and ensure the model's capability across diverse downstream tasks, it is specifically trained on the newly created Instruct (Pellegrini et al., 2023) dataset. This dataset is meticulously curated to encompass a spectrum of eight diverse tasks: RG, NLE, complete CheXpert QA, binary CheXpert QA, region QA, summarization, report correction, and reformulation report using simple language. Carefully formulated prompts accompany each task, tailored to elicit specific responses from the model. For instance, some prompts involve answering questions about particular X-ray regions. RaDialog trained on the Instruct dataset achieves an F1 score of 0.397 on the binary CheXpert QA task and 0.403 on the complete CheXpert QA task. In contrast, RaDialog without being trained on Instruct achieves lower F1 scores of 0.018 and 0.098, respectively.

#### 4.3.16 PathChat

PathChat is a multimodal generative AI copilot designed for human pathology (Lu et al., 2024b). It employs UNI (Chen et al., 2024), built on the ViT-L backbone and pre-trained using SSL on over 100 M histology image patches from approximately 100,000 WSIs, to generate visual features. PathChat uses the Llama 2 13B (Touvron et al., 2023b) LLM for text decoding, which is pre-trained on general text data. The UNI is connected to the LLM through a multimodal projector that maps visual tokens into the LLM's embedding space. During PathChat's pre-training phase, UNI and multimodal projector are trained on the CONCH (Lu et al., 2024a) dataset, comprising 1.18 M pathology image-caption pairs sourced from PMC-OA (Lin et al., 2023a) and internally curated datasets, aligning the image representations with pathology text while keeping the LLM weights frozen. The whole dataset is not publicly available. During instruction fine-tuning, the entire model is trained end-to-end on a specially curated PathChat dataset consisting of 456,916 pathology-specific instructions of 6 different types and 999,202 QA pairs. The model is evaluated on the newly curated PathQABench dataset, consisting of public and private subparts. On the multiple-choice questions across the entire PathQABench dataset, PathChat achieved an accuracy of 78.1% when only images and questions are provided to the model and 89.5% when clinical data is also included. For open-ended questions, PathChat attained an accuracy of 78.7% on the subset of questions for which pathologist evaluators reached a consensus.

## 5 Challenges and future directions

As VLMs become more prevalent in healthcare, various challenges and opportunities for future research emerge. This section highlights key obstacles and proposes research directions to improve VLM's effectiveness and seamless integration within clinical environments.

### 5.1 Data availability and privacy

A significant challenge in developing effective medical VLMs is the limited availability of ML-ready diverse and representative medical datasets. This limitation restricts the comprehensive training of VLMs, impeding their ability to understand the complexities of diverse and rare clinical scenarios (Moor et al., 2023). To mitigate privacy concerns, most datasets undergo rigorous pre-processing to remove Protected Health Information (PHI) before model training. The common approach is using algorithms to detect and remove sensitive information from structured and unstructured data. For example, Philter redacts PHI from clinical notes (Norgeot et al., 2020). ImageDePHI automates the removal of PHI from WSIs (Clunie et al., 2024). Another approach is replacing identifying information with artificial identifiers, which keeps data linkable without disclosing personal details. However, the risk of PHI leakage still remains a concern.

In the future, addressing this limitation can involve employing innovative approaches such as RAG and federated learning (FL). While RAG usually involves a frozen model during training, exploring the pre-training of VLMs within the RAG framework opens up a new avenue of research (Zhao et al., 2023). This innovative approach can potentially enhance the robustness of VLMs, especially in handling new and unforeseen medical cases. Additionally, FL offers a promising strategy to address data scarcity while protecting patient privacy (Zhang et al., 2021). In FL, models are trained locally at multiple institutions on their own patient data. Each institution shares the updated model weights with the central server. The server then aggregates these weights to create a global model. Later, the updated global model can be sent back to institutions for fine-tuning. To further safeguard privacy, the weights in FL can be protected using techniques such as differential privacy (DP) or homomorphic encryption (HE). In DP, noise is added to the gradients before they are sent to the central server (Dwork, 2006). In contrast, HE encrypts the weights, allowing the central server to perform computations on them without decryption (Stripelis et al., 2021). Future research can focus on optimizing the balance between privacy and performance of VLMs, and enhancing the efficiency of encryption methods in FL (Koutsoubis et al., 2024b,a).

### 5.2 Proper evaluation metrics

In medical RG, traditional metrics like BLEU and ROUGE can be used to effectively quantify surface-level linguistic similarity by capturing text overlap and structural matching between generated and reference texts. METEOR goes further by accounting for synonyms and stemming, providing a more nuanced view of textual similarities. Perplexity, often used to measure language fluency, evaluates how well the generated text adheres to natural language patterns. Together, these metrics assess fluency, coherence, and overall readability, ensuring that generated reports are well-formed and comprehensible. However, they often fall short in capturing the nuanced complexities of clinical language and contextual relevance critical in medical settings (Yu et al., 2023). Specifically, they may fail to determine whether a report accurately conveys essential clinical findings or diagnoses. Advanced metrics like BERTScore seek to assess semantic similarity beyond surface-level text overlap, but they require fine-tuning on medical datasets to understand specialized terminology and relationships, and may still miss subtle clinical nuances.

In medical VQA, traditional metrics like Accuracy, Precision, and Recall are commonly used to evaluate how well VLMs answer clinical questions, such as identifying medical conditions or anatomical features. While these metrics effectively assess binary outcomes, they fail to account for the varying clinical relevance or significance of errors made by the model. For example, misclassifying a serious condition may have far more severe consequences than making minor prediction errors, yet this distinction is not captured in simple accuracy-based evaluations.

To address the limitations of traditional metrics, it is imperative to develop specialized metrics tailored for medical RG and VQA, particularly for open-ended medical queries. For instance, RadGraph F1 (Yu et al., 2023) was developed to evaluate the extraction of clinical entities (e.g., diagnoses, findings) and their relations (e.g., linking conditions to anatomical locations) in radiology reports. This metric is particularly valuable for assessing structured medical data, ensuring that reports capture not only relevant clinical entities but also their correct relationships, which is crucial for the accuracy and integrity of medical conclusions. The development of additional specialized metrics is vital for evaluating VLMs performance and for assessing factors such as generalization, efficiency, and robustness in clinical decision-making and diagnostic support. Furthermore, integrating these metrics with other quantitative measures and human assessments can significantly enhance evaluations and drive continuous advancements in the capabilities of medical VLMs.

### 5.3 Hallucinations

The issue of hallucinations in generative VLMs poses a significant challenge to their reliability and practical application (Liu et al., 2024). Hallucinations refer to instances where VLMs generate outputs that are not grounded in the provided images or inconsistent with the established knowledge. In medical contexts, these hallucinations can have serious consequences, leading to inaccurate diagnostic information or treatment recommendations. One identified cause of hallucinations is the lack of alignment between visual and textual information (Sun et al., 2023). Training VLMs to effectively align these data modalities is crucial in mitigating the risk of hallucinations. For instance, LLaVA-RLHF (Sun et al., 2023) achieved hallucination reduction by incorporating RLHF to align different modalities. Further research can focus on integrating RLHF into medical VLMs. Additionally, incorporating RAG can help reduce the risk of generating misleading or fabricated outputs by allowing the system to analyze medical images while simultaneously accessing relevant information from trusted textual sources.

### 5.4 Catastrophic forgetting

Overcoming catastrophic forgetting poses an additional challenge in the development of medical VLMs. Catastrophic forgetting occurs when a model learns new information but inadvertently erases or distorts previously acquired knowledge, potentially compromising its overall competence. Striking a balance during fine-tuning can be crucial; moderate fine-tuning can be helpful to adapt the model to a specific task, while excessive fine-tuning can lead to catastrophic forgetting (Zhai et al., 2023; Khan et al., 2023). As a future research direction, leveraging methodologies from continual learning (Wang et al., 2023a; Zhou et al., 2023a; Cai and Rostami, 2024; Khan et al., 2023, 2024) might be useful in the context of medical VLMs. Continual learning focuses on training models to sequentially learn from and adapt to new data while retaining knowledge from previously encountered tasks (Khan et al., 2024). Also, incorporating adapters within the framework of continual learning can be a valuable tool in mitigating catastrophic forgetting (Zhang et al., 2023b).

### 5.5 Integration into hospital systems

Integrating VLMs into hospital systems also presents substantial challenges, requiring extensive collaboration between medical professionals and AI/ML researchers. First, medical professionals must maintain rigorous data collection practices to ensure that the data is clean, well-organized, and accessible, as ML experts rely on high-quality data to train and fine-tune VLMs. Second, VLMs must be designed to address the right clinical questions, ensuring their relevance and utility in medical practice. Third, healthcare professionals need training to use VLMs effectively, and the models should be intuitive and user-friendly to integrate smoothly into daily clinical routines. Furthermore, implementation scientists play a crucial role in this process by facilitating collaboration between clinicians and ML experts (Reddy, 2024). They help bridge the gap between these two groups, ensuring that VLMs are both technically robust and clinically relevant.

In this context, models like RaDialog (Pellegrini et al., 2023) and PathChat (Lu et al., 2024b) show the potential of VLMs to enhance clinical effectiveness. RaDialog demonstrates a solid capability to produce clinically accurate radiology reports. It transforms static reporting into a dynamic tool where clinicians can ask follow-up questions and seamlessly incorporate expert insights. This aligns closely with the interactive processes typical in clinical settings. Meanwhile, PathChat demonstrates promising clinical effectiveness as an AI copilot for pathology. It can assist pathologists in their work in real medical settings, including human-in-the-loop clinical decision-making, complex diagnostic workups, analyzing morphological details in histology images, and guiding immunohistochemistry (IHC) interpretations. However, the assessment of VLM effectiveness in medical environments is an open research question. Comprehensive clinical trials are necessary to confirm that VLMs truly enhance patient care and integrate effectively into existing clinical workflows.

### 5.6 Security

The security of VLMs must be thoroughly considered, focusing on privacy, minimizing medical errors, and preventing the introduction of significant new errors. VLMs must be kept behind the hospital firewall to protect sensitive medical information. It is also crucial to involve independent experts in the validation process. Validating the model on unseen medical data can help identify and rectify potential inaccuracies. Additionally, adversarial attacks represent another significant security issue, as they can exploit vulnerabilities in the model, leading to incorrect predictions. To combat this, incorporating adversarial training by exposing the model to adversarial examples during training can enhance its robustness against such attacks (He et al., 2023a). Continuous monitoring and updating of the VLMs are also essential to prevent the introduction of new errors, which should include regular audits and updates based on the latest medical research and clinical guidelines.

## 6 Conclusion

This review paper highlights the transformative potential of VLMs in generating medical reports and answering clinical questions from medical images. It explores 16 recent medical VLMs, among which 15 are publicly available. We observed that 6 of them share a similar architecture that has only recently become common. These VLMs incorporate a vision encoder, often with a projection module, to produce visual features, which can be used as input to LLMs. The visual features are then combined with tokenized text input and fed into the LLM. This approach simplifies model design and leverages the extensive prior knowledge embedded in LLMs. Furthermore, feeding all data features into LLMs enables the generation of human-like text outputs, improving user experience and facilitating more effective communication of medical insights. The review also explores 18 publicly available medical vision-language datasets and over 10 evaluation metrics for RG and VQA. By providing essential background information, this review ensures accessibility for readers from the medical field while promoting collaboration between the AI/ML community and medical professionals.

Moreover, the review highlights the current challenges and potential future directions for VLMs in medicine. The limited availability of diverse medical datasets and privacy concerns can be addressed through rigorous data pre-processing and techniques like RAG and FL. Also, since traditional evaluation metrics often fall short of capturing the nuances of clinical language, there is a need to develop specialized metrics tailored to medical RG and VQA. It is likewise crucial to address VLM hallucinations, and incorporating RLHF and RAG are promising solutions. Continual learning methods can help mitigate catastrophic forgetting, ensuring that models retain the knowledge they have previously acquired. Furthermore, collaboration between healthcare professionals and AI researchers is essential to deploy VLMs in ways that genuinely improve patient care. Lastly, ensuring the security of these models is vital, which can be achieved through robust firewalls and adversarial training. Ultimately, the review serves as a valuable resource for researchers developing and refining VLMs for medical applications, guiding them in overcoming key obstacles and leveraging innovative approaches to enhance model performance and clinical integration.
